# Highly Reversible Zinc Anode Enabled by a Thiourea‐Derived Protective Layer for Alkaline Zinc Batteries

**DOI:** 10.1002/advs.75929

**Published:** 2026-06-04

**Authors:** Noam Navon, Alagar Raja Kottaichamy, Thamaraichelvan Marichelvam, Jonathan Tzadikov, Michael Volokh, Liel Abisdris, Alexander Upcher, Menny Shalom

**Affiliations:** ^1^ Department of Chemistry Ben‐Gurion University of the Negev Beer‐Sheva Israel; ^2^ Academy of Scientific and Innovative Research (AcSIR) Ghaziabad India; ^3^ Electrochemical Power Sources Division CSIR‐CECRI Karaikudi Tamil Nadu India; ^4^ Ilse Katz Institute for Nanoscale Science and Technology Ben‐Gurion University of the Negev Beer‐Sheva Israel

**Keywords:** dendrite suppression, protecting layer, zinc anode, Zn–air batteries

## Abstract

Zinc‐based batteries hold great promise as next‐generation energy storage systems due to their low cost, intrinsic safety, and high energy density. However, their long‐term stability and efficiency are severely limited by the irreversible zinc plating/stripping reactions that lead to dendrite growth and parasitic side reactions, such as ZnO formation and hydrogen evolution. Here, we present a simple strategy to stabilize the zinc anode by growing a conjoint zinc sulfide (ZnS) and polymeric carbon nitride (CN) binder‐free protective layer directly on the Zn surface. Structural and electrochemical analyses demonstrate that this hybrid interphase effectively regulates Zn^2+^ nucleation, suppresses dendrite formation, and minimizes parasitic reactions, thereby enabling highly reversible zinc cycling with excellent long‐term stability. The optimized Zn anode exhibits a low voltage hysteresis of 170 mV at a high areal capacity of 30 mAh cm^−2^ (at 30 mA cm^−2^) for over 170 h, achieving more than three times the cycle life of bare zinc. When integrated into a rechargeable zinc–peroxide battery, it delivers state‐of‐the‐art performance, maintaining stable operation for 1100 h at 9 mAh cm^−2^.

## Graphical abstract

A ZnS/carbon nitride (ZnS/CN) protective coating is constructed on Zn anodes via ultrasonic spray coating, enabling uniform and stable Zn stripping/plating, suppressing dendrite formation, and mitigating parasitic side reactions, resulting in a prolonged cycling lifespan and high reversibility of the Zn anode in alkaline Zn–air batteries.

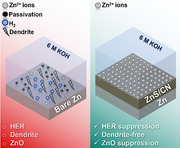

## 1 Introduction

The rapid development of electric vehicles, mobile technology, and grid‐scale storage solutions, coupled with growing concerns about fossil fuel depletion and environmental degradation, has created an urgent need for high‐performance, sustainable, efficient, reliable, and economically viable electrochemical energy storage devices [1, 2]. Zn‐based energy storage devices have emerged as promising candidates across various configurations, including zinc ion batteries, zinc–air batteries, and both aqueous and non‐aqueous zinc metal battery systems. This versatility can be attributed to the favorable properties of Zn electrodes, including low toxicity, intrinsic safety, low manufacturing cost, and high theoretical capacity of 820 mAh g^−1^ [3]. Additionally, zinc's low redox potential (−0.76 V vs. standard hydrogen electrode (SHE)) and significant overpotential for hydrogen evolution support a wide operational voltage range of approximately 1.8 V in zinc‐based batteries [3, 4]. However, despite these promising characteristics, the development of Zn‐based energy storage devices has been severely limited by fundamental challenges with the Zn‐based anode electrode, particularly in highly alkaline environments [5, 6]. These fundamental limitations are characterized by critical issues, including low Coulombic efficiency and limited cycle life due to uneven Zn stripping/plating processes, dendrite formation, and side reactions, including irreversible ZnO byproduct formation and the hydrogen evolution reaction (HER) [7, 8]. It is well established that Zn reversibility is significantly hindered by the parasitic HER in Zn metal‐based electrochemical devices, as the potential of the hydrogen evolution reaction (0 V vs SHE) is higher than that of Zn^2+^/Zn reduction (−0.76 V vs SHE) [9, 10].

Dendrite growth can penetrate the separator, causing internal short circuits that compromise battery safety, increase resistance, reduce efficiency, accelerate capacity loss, and ultimately shorten lifespan [11]. The persistent challenge of ensuring voltage reversibility while maintaining electrode stability in highly alkaline solutions has remained a significant barrier to practical applications such as reversible zinc–air batteries (RZABs) [12]. To overcome these challenges, considerable efforts have focused on enhancing the stability of Zn anode electrodes through Zn alloying, protective surface coatings, conductive additives, modified electrolyte compositions, and novel cell configurations [13, 14, 15, 16]. Among these strategies, surface modifications have shown great promise for addressing key limitations of zinc anodes by regulating the interactions between the zinc surface and the electrolyte. Specifically, interface modification with various coating materials, such as metal oxides [17, 18] polymer coatings [19], and graphene‐based materials [20], is a promising approach that directly addresses dendrite formation and side reactions [21]. These types of coatings facilitate the uniform distribution of zinc ions during electrodeposition and enhance the reversibility of zinc redox cycling processes [22, 23]. Despite significant progress in this field, substantial challenges remain in developing a uniform coating layer on Zn anode electrodes that balances adequate thickness for protection with sufficient ionic permeability for electrochemical performance, particularly in a highly alkaline environment. Recently, ZnS has garnered significant attention as a protective interface material for zinc anodes [24, 25, 26] serving as a physical barrier on the Zn surface. This protection is attributed to its chemical inertness, excellent stability in aqueous electrolytes, and high selectivity for zinc ions [27]. Additionally, the unbalanced charge distribution of ZnS promotes rapid Zn^2+^ transport at the interface, facilitating uniform Zn stripping/plating [19].

Herein, we report a simple, efficient, cost‐effective, and binder‐free strategy for fabricating a conjoint ZnS and polymeric carbon nitride (CN)‐based protective layer on the surface of the Zn electrode, using an ultrasonically deposited thiourea precursor followed by thermal condensation, forming a Zn/ZnS/CN structure. This layer introduces a physical barrier between the highly alkaline electrolyte and zincophilic sites that regulate Zn^2+^ nucleation [28].

The best‐performing and optimized protected Zn anode exhibits a low voltage hysteresis vs Zn of 170 mV at an areal capacity of 30 mAh cm^−2^ (30 mA cm^−2^) for more than 170 h in 6 M KOH(aq). This increased stability is attributed to the successful mitigation of parasitic reactions, including hydrogen evolution, dendrite formation, and ZnO formation, which demonstrates the effectiveness of the protective layer. This results in uniform Zn deposition, which, in turn, enhances the Zn anode's reversibility, cycling lifetime, and overall electrochemical performance. As a proof of concept, the optimized protected Zn anode was used in a rechargeable Zn–peroxide battery (ZPB) at 9 mAh cm^−2^ for more than 1100 h, showing an almost threefold improvement in cycling lifetime compared to an unprotected commercial Zn anode, while exhibiting an exceptional round‐trip energy efficiency of 92% ± 3%.

## 2 Results and Discussion

### 2.1 Preparation and Characterization of pZn_x_ Electrodes

**FIGURE 1:**
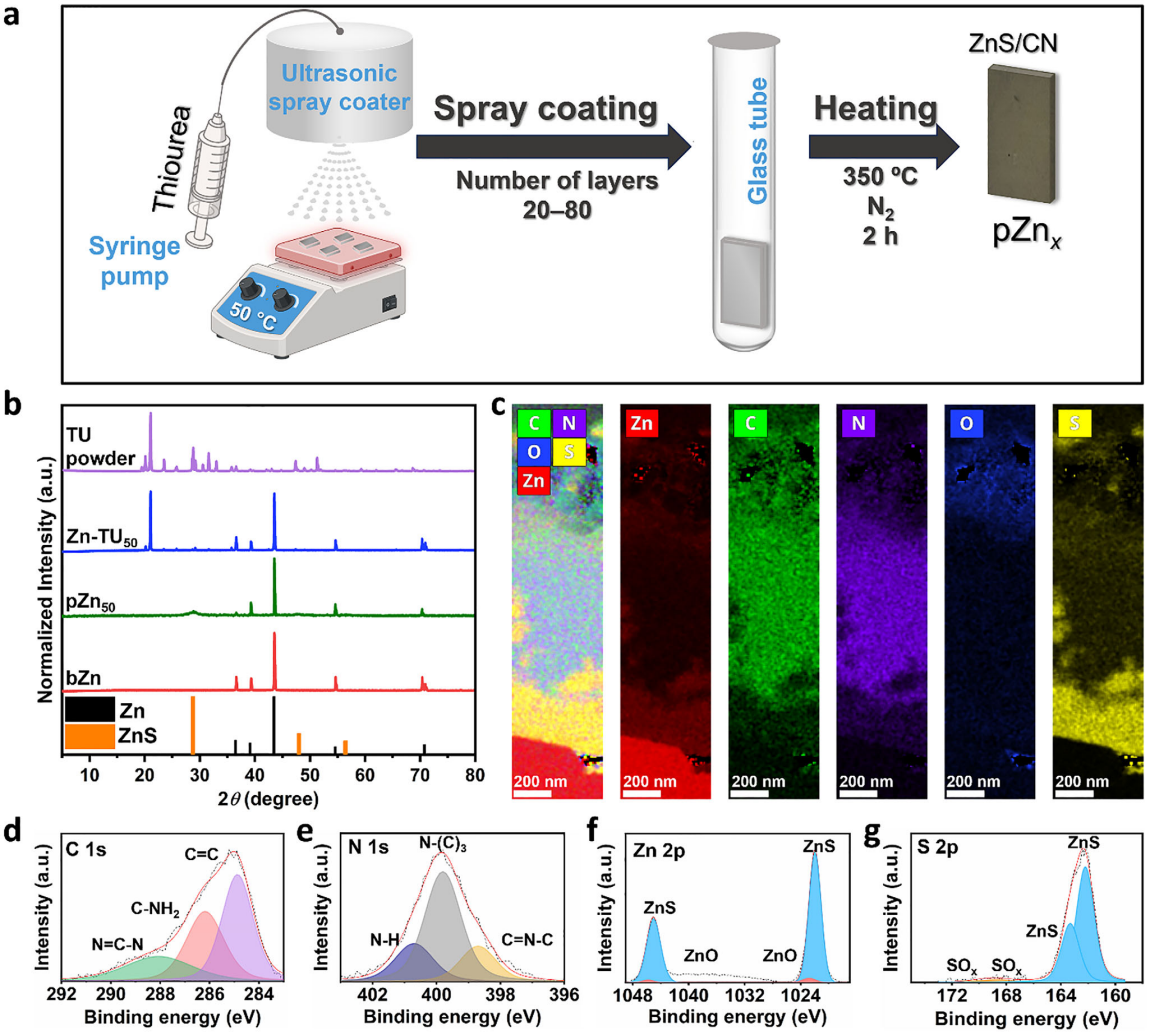
Structural analysis of pZn*_x_* electrodes. (a) Schematic illustration of the synthesized protective layer on a zinc electrode surface. (b) XRD patterns of the thiourea precursor powder (TU), the resulting coated Zn sheets (Zn‐TU_50_), pZn_50_, and a bZn sheet; bZn, pZn_50_, and Zn‐TU_50_ are normalized to Zn's diffraction signal ca. 43.47°, corresponding to Zn (101) plane. The TU powder pattern is normalized to its strongest diffraction ca. 21.1°. The patterns are vertically offset for clarity. (c) High‐angle annular dark field (HAADF) STEM and EDS elemental mappings of pZn_50_. (d) C 1s, (e) N 1s, (f) Zn 2p, and (g) S 2p XPS spectra of pZn_50_.

The synthetic route toward protected zinc electrodes is illustrated in Figure 1a and was adapted from a previously developed procedure in our group for the deposition of polymeric carbon nitride over transparent conductive electrodes using ultrasonic spray coating of suitable precursors [29, 30]. Briefly, uniform seeding layers of thiourea (TU) precursor were deposited on zinc foil substrates heated to 50 °C over a hot plate, while controlling layer thickness through altering the number of spray cycles (20, 40, 50, or 80) of an aqueous TU solution (125 g L^−1^). Our hypothesis was that by varying precursor deposition cycles, we could control the thickness and coverage of the protective layer to find an optimum that provides protection without hampering electrochemical performance, as detailed later. Subsequently, the precursor‐coated zinc foils (Zn‐TU*_x_*, where *x* stands for the number of spray cycles) underwent thermal treatment at 350 °C under an N_2_ atmosphere for two hours, resulting in protected zinc electrodes (denoted as pZn*_x_*). The synthesis process involves the thermal decomposition of thiourea, with H_2_S release in the range of 180°C–220 °C [31], providing sulfide intermediates that react with Zn to form ZnS [32].

Thiourea is particularly suitable as a sulfur precursor due to its gradual decomposition at relatively low temperatures [33], which enables controlled film formation below the Zn melting point (419.5 °C) [34]. In addition to serving as a sulfur source, thiourea decomposes at 350 °C to release carbon and nitrogen species, leading to partial carbon nitride polymerization, as the reaction temperature is limited by the melting point of Zn. This yields nitrogen‐rich carbonaceous structures with C─N bonds and zincophilic sites such as *s*‐triazine rings, ─NH_2_, and ─CN groups; this polymeric material is referred to herein as CN. CN's nitrogen‐containing moieties serve as coordination sites to Zn^2+^(aq), thus regulating nucleation, suppressing parasitic reactions, and forming robust layers that protect the Zn's surface during cycling [35]. To verify the formation of ZnS/CN coatings and optimize the layer thickness, structural and chemical analyses were performed using scanning electron microscopy (SEM), x‐ray diffraction (XRD), and Fourier‐transform infrared (FTIR) spectroscopy on all coated samples (pZn_20–80_, where 20–80 represents the range of applied TU precursor spray cycles).

A thicker protective coating can enhance physical isolation between the zinc surface and the electrolyte, improving protection against corrosion and parasitic reactions. However, excessive thickness may hinder Zn^2+^ ion transport and reduce electrochemical efficiency. In contrast, thinner coatings facilitate easier ion migration but may lack sufficient surface coverage, thereby limiting their effectiveness in suppressing side reactions and dendrite growth. Thus, identifying an appropriate coating thickness is crucial to balance ionic conductivity with interfacial stability. Top‐view and tilted‐view SEM analysis was conducted to examine the surface morphology and coating thickness across all samples (Figure S1), revealing differences in surface features and coating coverage. The pZn_80_ electrode formed a dense layer that may be too thick, potentially impeding Zn^2+^ ion migration at the electrode interface, as shown later. In contrast, the pZn_20_, pZn_40_, and pZn_50_ electrodes exhibited thinner surface layers that visibly cover the zinc surface. Tilted‐view images (Figure S2) demonstrate the expected trend, where an increasing coating thickness results from a larger number of deposited layers (*x*), while providing further insight into the overall interface quality.

The pZn_20_ electrode displayed a very thin, non‐uniform coating with weak adhesion to the zinc substrate, indicating insufficient coverage and limited protection. The pZn_80_, on the other hand, exhibits an excessively thick coating with inconsistent thickness across the interface, suggesting poor uniformity and potential interfacial instability. However, the pZn_40_ and pZn_50_ electrodes show good uniformity and continuous coatings of moderate thickness, which may provide a favorable balance between ionic conductivity and surface protection. To complement the SEM analysis and quantitatively assess coating thickness, all pZn*_x_* electrodes were analyzed using a 3D laser‐scanning confocal microscope (LEXT). The measured thicknesses for pZn_20_, pZn_40_, pZn_50_, and pZn_80_ were 1.25, 1.97, 2.18, and 3.85 µm, respectively, confirming that coating thickness increases with the number of deposition cycles (Table S1). These quantitative measurements correlate with the tilted‐view SEM analysis (Figure S2), which revealed distinct morphological differences consistent with the varying coating thicknesses among the electrodes.

The XRD pattern (Figure 1b) of Zn‐TU_50_ exhibits identical diffraction features to those of bare Zn (bZn) and pristine TU powder, indicating that thiourea has successfully and intact coated Zn before thermal treatment. Upon thermal treatment, XRD was conducted for all coated samples (Figure S3a). Distinct diffraction peaks of cubic ZnS (i.e., a zinc blende phase) were observed at ∼28.8° and ∼47.9° (corresponding to the (111) and (220) planes), predominantly in the pZn_50_ and pZn_80_ samples, with the most intense reflections observed for pZn_50_ [36]. A broad peak centered at ∼27.6°, corresponding to the interlayer stacking (002) plane, is most pronounced in the pZn_50_ sample, indicating successful partial CN polymerization [37]. The close proximity of this peak and the ZnS (111) peak at ∼28.8° results in a merged broad feature, as further evidenced in the magnified view of the 2*θ* = 20°–36° region (Figure 3b), where both peaks are identified across all electrodes, further highlighting pZn_50_ as the sample with the most evident ZnS/CN formation [38]. The emergence of ZnS and CN signals supports the transformation of the thiourea layer into a ZnS/CN composite over the Zn substrate. FTIR analysis of all coated electrodes (Figure S4) confirms this analysis by revealing bands corresponding to cyano (─CN) and amino (─NH_2_) groups, as well as characteristic vibrations of *s*‐triazine structures, indicative of at least partial condensation into a polymeric carbon nitride for all samples.

Among all the protected electrodes, pZn_50_ exhibited the best electrochemical performance (which will be elaborated in detail later; see, for example, the rate profiles of a symmetric Zn||Zn cell in Figure S5). As such, it was chosen for further characterization. To accurately evaluate the thickness and structure of the coated electrode, a focused ion beam (FIB) was used to prepare a cross‐sectional lamella of the pZn_50_ sample for high‐resolution scanning transmission electron microscopy (STEM) and energy‐dispersive X‐ray spectroscopy (EDS) analysis. The STEM image with overlaid EDS mapping (Figure 1c) reveals a clear three‐layer structure: a metallic Zn bottom layer, an intermediate ZnS layer, and a top layer composed of carbon and nitrogen, attributed to the polymeric CN coating. The atomic fraction profile and the corresponding elemental mapping (Figure S6a,b, respectively) confirm this structure, revealing a Zn‐rich bottom layer, an intermediate region where S and Zn atoms are concentrated, and an upper layer dominated by C and N. Within this structure, localized ZnS penetration into the upper region of the CN top layer is observed (Figure S6c), as indicated by the lighter gray regions (characteristic of the ZnS phase). The STEM analysis therein reveals that the individual layer thicknesses are approximately 0.45–0.50 µm for the ZnS intermediate layer and ∼0.75–1.00 µm for the CN top layer. To further support these findings, a cross‐sectional SEM analysis of pZn_50_ (Figure S7) revealed a thickness range of 1.3–2.8 µm, consistent with the LEXT (2.18 µm) and STEM (∼1.3 µm) measurements. The slight variation may be attributed to partial coating detachment during sample preparation for cross‐sectional SEM and STEM analysis. Additional structural evidence for ZnS formation was obtained by selected area electron diffraction (SAED) (Figure S8). The diffraction pattern from the coating layer shows three distinct rings corresponding to the (111), (220), and (311) planes of cubic ZnS [39].

The X‐ray photoelectron spectroscopy (XPS) after etching (i.e., measuring inside the coating layer and not the surface) of C 1s, N 1s, Zn 2p, and S 2p for pZn_50_ is shown in Figure 1d–g. The C 1s spectrum displays three peaks at 284.8, 286.1, and 288.5 eV, corresponding to C═C, C─NH_2_, and N═C─N, respectively [40, 41]. The N 1s spectrum includes peaks at 398.7, 399.8, and 400.7 eV, assigned to C═N─C, N─ (C)_3_, and N─H species [40, 42, 43, 44]. The Zn 2p spectrum exhibits two peaks at 1022.0 and 1045.1 eV, corresponding to the Zn 2p_3/2_ and Zn 2p_1/2_ transitions of ZnS [45]. Additionally, weak ZnO‐related peaks were observed in the Zn 2p spectrum at 1022.8 and 1045.8 eV, likely originating from slight surface oxidation of the zinc substrate during thermal treatment [46]. The S 2p peaks at 162.2 and 163.3 eV correspond to S 2p_3/2_ and S 2p_1/2_ of ZnS, respectively [47]. The weak S 2p peaks at 169.4 and 170.5 eV correspond to S 2p_3/2_ and S 2p_1/2_ of SO*_x_* species, respectively [48]. Notably, the XPS depth profile (Figure S9) confirms the presence of both CN and ZnS species throughout the probed depth over the entire etching duration, consistent with the STEM–EDS analysis. To further investigate the formation mechanism of the ZnS/CN coating, XRD measurements were conducted on pZn_50_ electrodes synthesized at various temperatures (100°C, 200°C, 300°C, 350°C, and 450 °C) (Figure S10). Among the tested conditions, 350 °C was identified as the optimal annealing temperature for ZnS/CN formation (see full discussion in the SI).

As the pZn_50_ synthesis produces uniform coatings with high reproducibility (supported by the top‐view SEM images in Figure S11), and based on the combined morphological and chemical analysis, pZn_50_ was determined to exhibit the optimal coating thickness, potentially offering favorable conditions for both Zn^2+^ ion transport and interfacial protection, which are key factors in mitigating parasitic reactions and dendritic growth.

### 2.2 Electrochemical Evaluation of Various Zn Electrodes in Symmetric Zn||Zn Half‐Cells

To evaluate the performance of pZn*_x_* electrodes with varying coating thicknesses (ranging from 20 to 80 deposition cycles; Figure S5, see the experimental section (Supporting Information) for details), we conducted electrochemical studies in a symmetric cell (Zn||Zn) configuration. Zn stripping/plating (Zn reversibility) was assessed at current densities ranging from 5 to 30 mA cm^−2^, at an areal capacity of 5 mAh cm^−2^.

**FIGURE 2:**
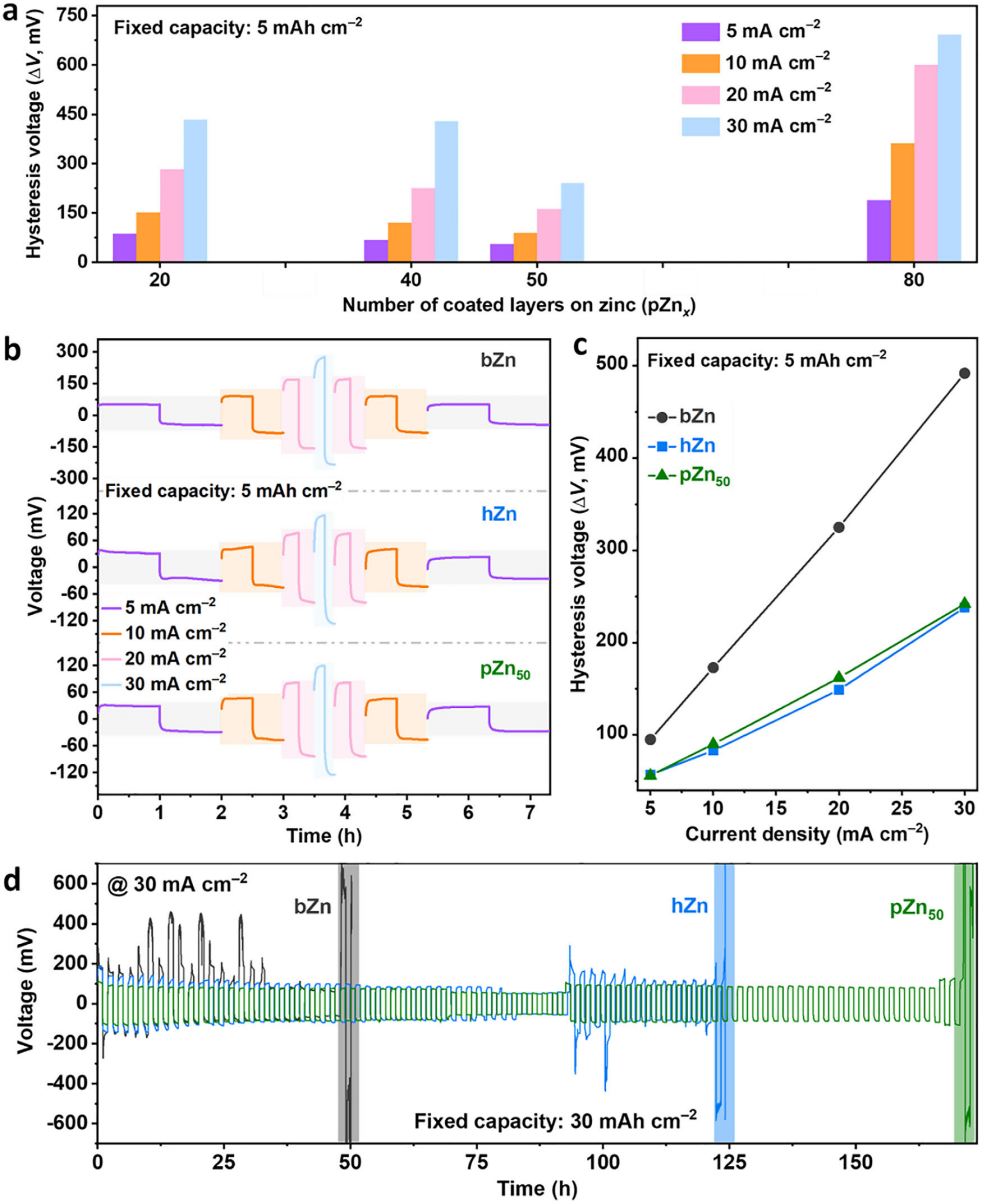
Electrochemical evaluation of various Zn electrodes. (a) Voltage profiles of pZn*_x_* electrodes with varying TU coating layers (*x* = 20, 40, 50, and 80) at current densities ranging from 5 to 30 mA cm^−2^, with a fixed areal capacity of 5 mAh cm^−2^. Comparative studies of non‐heated bare Zn (bZn), heated bare Zn (hZn), and pZn_50_ electrodes: (b) rate performance comparison under current densities of 5–30 mA cm^−2^ at a constant capacity of 5 mAh cm^−2^, (c) hysteresis voltage vs applied current density, and (d) long‐term cycling performance of Zn plating/stripping at a high current density of 30 mA cm^−2^ with a fixed areal capacity of 30 mAh cm^−2^ in symmetric Zn||Zn cells.

The pZn_50_ electrode shows the smallest hysteresis voltage gap across various current rates among all pZn*_x_* electrodes (Figure 2a and Figure S5). This superior performance is consistent with the optimized structure of pZn_50_, characterized by full surface coverage and the formation of a distinct ZnS/CN protective layer, as discussed in the previous section. The smallest hysteresis voltage gap indicates improved Zn^2+^ ion transport kinetics and more uniform electrodeposition, both of which are linked to a longer cycling lifespan [49]. Due to its high stability and minimal hysteresis, pZn_50_ was selected for further electrochemical testing.

To elucidate the effects of thermal treatment, a bZn electrode was also subjected to the same thermal conditions without TU layer coating. Figure 2b shows the comparative voltage gap profiles at different current rates for non‐heated bZn, heated bare Zn (hZn), and pZn_50_ electrodes. The bZn electrode exhibits a significantly larger hysteresis voltage gap than hZn and pZn_50_ electrodes. Notably, as the current density increases, the voltage gap of bZn rises sharply, further widening the difference compared to the other two electrodes (Figure 2c). These differences may arise from changes in surface morphology caused by thermal treatment (with or without coating), which likely improved Zn surface characteristics compared to bare Zn and enhanced electrochemical performance [50]. The surface morphology of Zn critically affects its stripping/plating performance. Smooth surfaces promote uniform Zn^2+^ deposition and suppress dendrite formation by limiting nucleation at surface irregularities and reducing local electric field intensity [51]. In contrast, rough interfaces enhance local ion flux, leading to uneven Zn growth. Additionally, reduced surface roughness mitigates side reactions such as HER, contributing to improved interfacial stability [52]. This correlation between morphology and electrochemical performance will be further examined in the following sections through SEM analysis.

To evaluate the practical applicability and effectiveness of the coating layer under high‐capacity and long‐term cycling conditions, we examined the electrochemical performance of bZn, hZn, and pZn_50_ in symmetric cells at a high current density of 30 mA cm^−2^ and a fixed areal capacity of 30 mAh cm^−2^ (Figure 2d). Among all tested electrodes, pZn_50_ exhibited the best Zn^2+^/Zn reversibility, with a prolonged cycling lifespan of 170 h, compared to 50 h for bZn and 120 h for hZn, and a significantly reduced voltage hysteresis of 170 mV, in contrast to 365 mV for bZn and 211 mV for hZn. Without the synergistic role of the conjoint ZnS/CN structure, Zn electrodes coated with either CN (Zn@CN) or ZnS (Zn@ZnS) (see, Section S4 and Figures S12–S14 for synthetic details, characterization, and electrochemical performance) exhibited pronounced voltage fluctuations and multiple electrical disturbances, indicating that dendrite growth initiates earlier and progresses more extensively when only ZnS or CN is present. These results confirm that the conjoint ZnS/CN structure is essential for effective dendrite suppression and uniform Zn stripping/plating. Notably, the extended cycling lifespan of 170 h achieved by pZn_50_ under these harsh conditions, together with its low voltage hysteresis, outperformed previously reported Zn anodes tested under identical parameters, including zwitterionic molecule‐modified Zn (110 h, 200 mV) [53], and in situ polydopamine solid electrolyte interphase (SEI)‐protected Zn (78 h, 200 mV) [54].

Further comparisons to other Zn metal anodes tested in symmetric Zn||Zn cells at high current densities and areal capacities are provided in Table S2, highlighting the high performance enabled by the ZnS/CN protective coating. The voltage profiles also highlight the enhanced stability of pZn_50_. Unlike bZn, which exhibited highly unstable profiles with fluctuating plating/stripping behavior, pZn_50_ maintained consistent voltage hysteresis throughout cycling. Although thermal treatment alone improved stability relative to bZn, pZn_50_ outperformed both bZn and hZn, delivering greater reversibility, lower voltage hysteresis, and enhanced durability. These findings underscore the effectiveness of combining thermal treatment with ZnS/CN surface coating to produce stable, high‐performance Zn‐based electrodes. Guided by its high performance, pZn_50_ was selected for extended cycling tests at a current density of 2 mA cm^−2^ and an areal capacity of 10 mAh cm^−2^ to further assess its long‐term durability. As shown in Figure S15, the pZn_50_ electrode exhibits stable cycling for ∼2000 h with a consistently stable voltage gap, demonstrating improved performance relative to state‐of‐the‐art Zn||Zn symmetric cells (Table S3).

### 2.3 Parasitic Chemistry Studies and Zn–Peroxide Battery (ZPB) Performance

**FIGURE 3:**
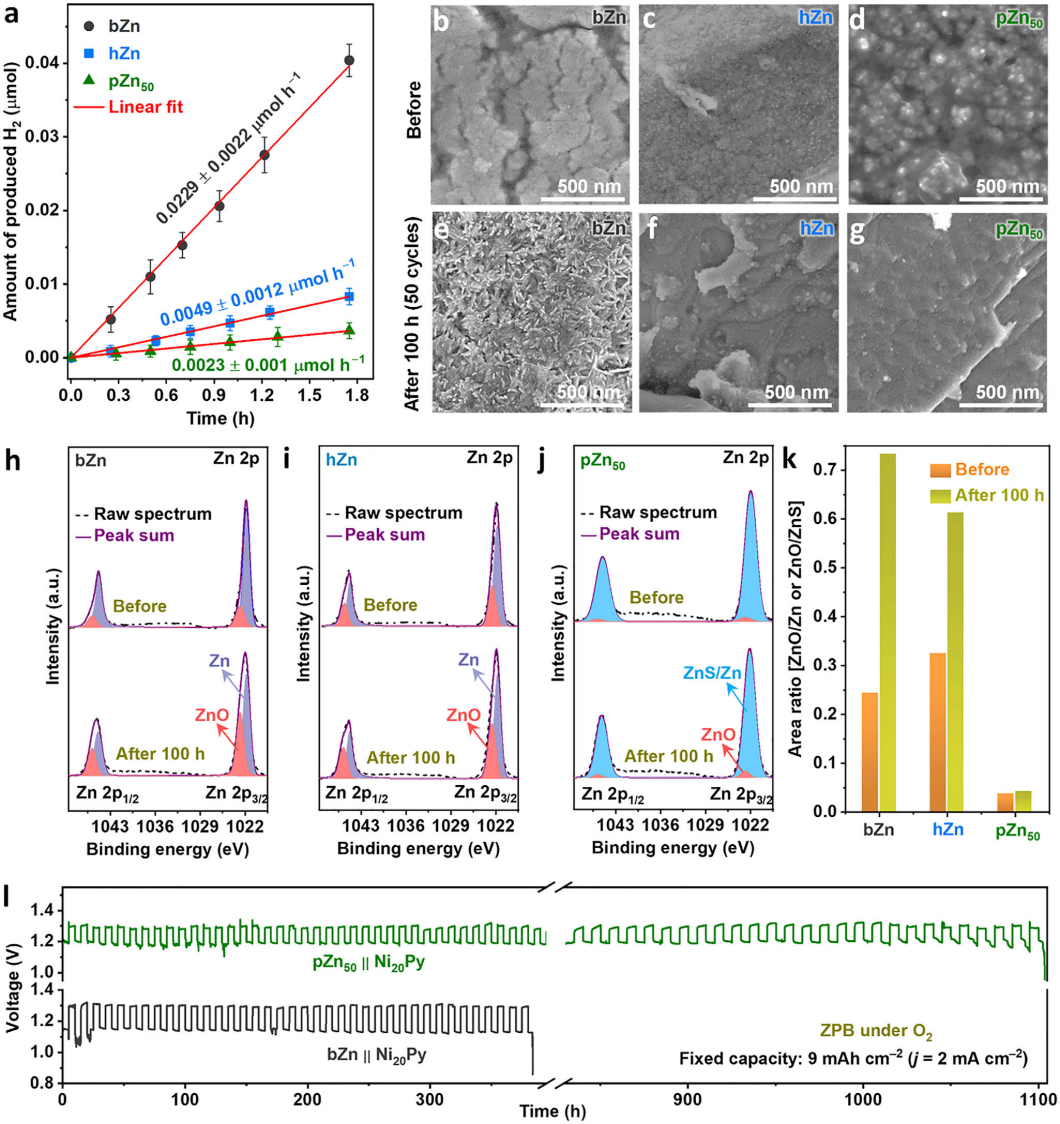
Parasitic reaction analysis and Zn–air battery performance. (a) Quantification of hydrogen gas evolution over time in the anodic compartment for bZn, hZn, and pZn_50_ electrodes; the linear fit corresponds to a constant (0th order) hydrogen production rate. (b–g) Top‐view SEM images of bZn, hZn, and pZn_50_ surfaces before and after a 100 h cycling test. (h–j) Comparative XPS spectra of bZn, hZn, and pZn_50_ electrodes before and after the 100 h cycling test. (k) ZnO/Zn or ZnO/ZnS area ratio extracted from the Zn 2p_3/2_ region in panels h–j. (l) Cycling performance of a two‐electrode Zn–peroxide battery employing Ni_20_Py as a bifunctional air cathode catalyst and pZn_50_ (or bZn) as the anode, tested at a fixed areal capacity of 9 mAh cm^−2^ and current density of 2 mA cm^−2^.

As discussed previously, for RZABs to realize their full potential, there is a strong need to address parasitic reactions that occur during Zn anode operation, including dendrite formation, anode passivation via ZnO deposition, and HER. To investigate the mitigation of Zn corrosion and resistance to parasitic HER, we performed Tafel analysis as shown in Figure S16 and H_2_ gas quantification using gas chromatography (GC) in Figure 3a, along with SEM and XPS analyses (Figure 3b–g,h–k) on bZn, hZn, and pZn_50_ electrodes before and after Zn reversibility tests. The Tafel polarization plots support the enhanced corrosion resistance, with a shift to positive potential from bZn (−1.41 V) to pZn_50_ (−1.38 V) [55, 56, 57]. This reduced corrosion rate and increased hydrogen overpotential is supported by H_2_(g) quantification by GC, which is carried out in parallel during the Zn reversibility cycling process at a current density of 2 mA cm^−2^ and areal capacity of 5 mAh cm^−2^ (see, Section S5, for details). Figure 3a shows that the pZn_50_ electrode produced the lowest amount of hydrogen in the anodic compartment during the Zn reversibility test (2.3 nmol h^−1^). The hZn electrode also exhibited a notable reduction in H_2_ evolution (4.9 nmol h^−1^) compared to the bZn electrode (22.9 nmol h^−1^), but its suppression effect was less pronounced than that of pZn_50_, indicating that the ZnS/CN protective coating layer contributed more effectively to HER mitigation than thermal treatment alone.

Next, the surface morphology of all electrodes was analyzed using SEM before and after a 100 h Zn^2+^/Zn reversibility test (50 cycles) in symmetric cells at a fixed current density of 1 mA cm^−2^ (corresponding to 1 mAh cm^−2^). Top‐view SEM images (Figure 3b–d) of the bZn, hZn, and pZn_50_ electrodes before cycling highlight morphological differences in their surfaces. The bZn surface appears rough and uneven (Figure 3b), while the hZn (Figure 3c) displays reduced surface roughness. In contrast, the pZn_50_ electrode (Figure 3d) features a dense, uniform coating that completely covers the Zn foil surface.

After 100 h of cycling tests, the bZn (Figure 3e) develops numerous sharp dendritic structures, resulting in a highly uneven surface. These dendrites can cause capacity degradation, internal short circuits, and reduced electrode lifespan [58]. In addition, they enlarge the electrode–electrolyte interfacial area, creating abundant reactive sites that locally reduce current density and thereby accelerate unwanted side reactions, such as hydrogen evolution and corrosion [10]. In contrast, the hZn (Figure 3f) and pZn_50_ (Figure 3g) maintain smooth, dendrite‐free surfaces, indicating high Zn^2+^/Zn reversibility and improved structural integrity during cycling, even under highly alkaline conditions, which are a known challenge for Zn–air batteries [59]. The observed morphological improvements, particularly the smooth, dendrite‐free surfaces of the hZn and pZn_50_ electrodes, are closely associated with reduced HER activity, as discussed in the previous section. The pZn_50_ electrode demonstrated a substantially reduced HER rate relative to both bZn and hZn electrodes. This improvement is attributed to the ZnS/CN protective layer, which effectively minimizes hydrogen bubble adhesion to the zinc surface during the HER process. The adherence of hydrogen bubbles to the electrode surface impedes the formation of proper nucleation sites, resulting in non‐uniform zinc deposition and surface roughening. These morphological changes subsequently accelerate dendrite formation and growth [10, 60]. While thermal treatment also lowers HER in hZn relative to bZn, leading to a smoother surface, the HER suppression in pZn_50_ is more pronounced and will maintain dendrite‐free morphology over extended cycling. These findings indicate the improved performance of the pZn_50_ electrode, where effective HER suppression and smooth morphology mutually reinforce each other, enhancing electrochemical stability and cycling performance (Figure 2d) in Zn‐based electrochemical storage.

To further investigate the surface chemical states of the electrodes, XPS was performed on bZn, hZn, and pZn_50_ electrodes before and after a 100 h Zn^2+^/Zn reversibility test (50 cycles) conducted in symmetric cells at a fixed current density of 1 mA cm^−2^ at an areal capacity of 1 mAh cm^−2^ (Figure 3h–j). Before cycling, both bZn and hZn exhibited characteristic Zn 2p_3/2_ metallic peaks at ∼1021.8 eV, along with noticeable ZnO signals at ∼1022.6 eV [61]. The pZn_50_ electrode displayed an additional distinct ZnS peak, consistent with the sulfur‐based surface modification previously discussed in Figure 1f,g. After 50 cycles, the bZn electrode showed a significant increase in the ZnO peak intensity (∼1022.75 eV), indicating the formation of a thick passivation layer that likely obstructs electrochemically active sites of the Zn anode (Figure 3h).

In comparison, the hZn electrode (Figure 3i) also showed ZnO growth, but to a lesser extent. The pZn_50_ (Figure 3j) demonstrated only a minimal increase in ZnO signal intensity, suggesting effective suppression of an irreversible ZnO layer formation during cycling. The higher contribution of ZnO in the bZn electrode is further confirmed by the ZnO/Zn or ZnO/ZnS ratio shown in Figure 3k. For bZn, this ratio increased by ∼200% after the cycling test, indicating substantial ZnO formation. In comparison, the hZn electrode showed a more moderate increase of ∼90%. Most notably, the ZnO/Zn or ZnO/ZnS ratio for the pZn_50_ electrode increased by only ∼10%, which is approximately three times lower than the increase observed in bZn, indicating the protective effect of the TU‐derived coating on the Zn surface. It suppresses the formation of irreversible ZnO layers, maintains electrochemical activity, and extends electrode lifespan in alkaline environments.

The surface wettability of the anodes was evaluated by contact angle measurements in a 6 M KOH + 0.20 M Zn(OAc)_2_ electrolyte. As shown in Table S4, the contact angles for the bZn, hZn, and pZn_50_ electrodes were 79°, 84°, and 44°, respectively. The significantly lower contact angle observed for the pZn_50_ electrode indicates enhanced surface wettability under the experimental conditions, thereby promoting favorable electrode–electrolyte interactions. Improved wettability is critical for achieving superior Zn^2+^/Zn reversibility, as it facilitates homogeneous Zn^2+^ ion distribution across the electrode surface, promoting uniform zinc nucleation that results in smooth plating and stripping behavior, thereby mitigating dendrite formation [62, 63, 64, 65]. Moreover, the enhanced wettability provides additional benefits by reducing hydrogen bubble adhesion to the electrode surface, thereby promoting stable and reversible zinc electrodeposition [66]. It also increases electrode–electrolyte contact area, boosting ionic transport across interfacial layers, and reducing charge transfer resistance, while decreasing interfacial free energy to improve ion transfer dynamics [67, 68].

To evaluate the practical applicability of the pZn_50_ electrode, we assembled a reversible, high‐energy‐efficiency alkaline Zn–peroxide battery (ZPB) system (cell structure shown in Figure S17). The battery delivers an open‐circuit voltage of 1.3 V with a peak power density of 128 mW cm^−2^ at a peak current density of 205 mA cm^−2^, by dissolution of Zn to Zn^2+^ at the anodic side and O_2_ reduction to peroxide as the cathodic half‐cell reaction (Figure S18) [12, 69]. Galvanostatic charge–discharge profiles for bare Zn and pZn_50_ were obtained at a current density of 2 mA cm^−2^ and a fixed areal capacity of 9 mAh cm^−2^ [12]. A custom‐made H‐cell employed a bifunctional Ni_20_Py catalyst as the cathode, which effectively facilitates both the 2e^−^ oxygen reduction reaction (ORR) to HO_2_^−^ and the peroxide oxidation reaction (POR) [12]; bZn (or) pZn_50_ electrodes serve as the anode (see Section S6, for battery assembly details). An anion‐conducting membrane was used to separate the anode and cathode. This configuration allows evaluating the Zn anodes without facing round‐trip efficiency issues [12] of traditional Zn–air batteries, where 4e^−^ redox electrocatalysts are required.

The ZPB configured with a bare Zn anode (bZn||Ni_20_Py) failed after approximately 380 h of operation (Figure 3l, gray line). In contrast, the ZPB using pZn_50_ anode (pZn_50_||Ni_20_Py) delivered exceptional durability, over 1100 h, which is nearly three times longer than its bZn counterpart (Figure 3l, green line). The voltage fluctuations observed in the pZn_50_||Ni_20_Py cycling curve at 50–150 h are attributed to transient oxygen bubble accumulation at the air cathode, arising from O_2_ released during peroxide oxidation combined with continuous oxygen flow to the cathode side, which periodically blocks active catalyst sites, with the voltage stabilizing beyond 150 h as bubbles gradually detach from the electrode surface. Round‐trip energy efficiency (*η*), which is limited by several factors, including non‐uniform Zn plating, dendrite formation, and corrosion at the Zn anode, was calculated as the ratio between the average discharge and charge voltages at a fixed capacity (see Supporting Information, Section S6, for details) [70, 71, 72]. Based on this calculation, the pZn_50_‐based ZPB exhibited a significantly higher and more stable *η* of 92% ± 3%, compared to 84% ± 2% for the bZn‐based system, throughout the entire cycling period, demonstrating improved performance relative to state‐of‐the‐art Zn–air battery cells Table S5). These performance improvements are attributed to the TU‐derived protective layer, which promotes uniform Zn deposition and effectively suppresses parasitic reactions such as hydrogen evolution. It also limits Zn corrosion and minimizes the continuous formation of irreversible ZnO during redox cycling under highly alkaline conditions. Together, the electrochemical study and ZPB performance results confirm that the TU‐derived protective layer on pZn_50_ anode delivers excellent stability, efficiency, and longevity, establishing it as a promising candidate for next‐generation high‐alkaline energy storage systems.

## 3 Conclusion

In this work, we present a simple and cost‐effective strategy to significantly enhance the performance and stability of Zn anodes via a thiourea‐derived protective coating. This method forms a homogeneous binder‐free ZnS/polymeric CN composite layer on the Zn foil surface. The optimized pZn_50_ electrode, featuring 50 coating layers, exhibited the lowest hysteresis voltage gap across all current densities in symmetric Zn||Zn configurations and demonstrated excellent stability, operating at a fixed areal capacity of 30 mAh cm^−2^ for 170 h in a highly alkaline 6 M KOH electrolyte. The ZnS/CN protective layer plays a critical role in regulating Zn^2+^ ion transport, effectively suppressing dendritic growth, parasitic hydrogen evolution, zinc corrosion, and irreversible ZnO formation during redox cycling under highly alkaline conditions. The protective coating leads to excellent cycling performance and long‐term stability of Zn anodes.

The real‐world applicability of pZn_50_ was validated in a reversible high‐efficiency alkaline Zn–peroxide battery (ZPB), where it served as the anode in combination with a nickel‐based bifunctional cathode. The ZPB incorporating pZn_50_ achieved a threefold increase in operational lifespan (∼1100 h) at 9 mAh cm^−2^ capacity compared to an untreated (bZn‐based) system (∼380 h), while maintaining a high round‐trip energy efficiency of 92% ± 3%. Overall, the new Zn/ZnS/CN configuration offers a robust and scalable route for developing stable Zn anodes, positioning pZn_50_ as a highly promising candidate for next‐generation alkaline aqueous energy storage systems.

## Conflicts of Interest

The authors declare no conflicts of interest.

## Supporting information




**Supporting File**: advs75929‐sup‐0001‐SuppMat.pdf.

## Data Availability

The data that support the findings of this study are available from the corresponding author upon reasonable request.
