# Supplementary material for: Highly Reversible Zinc Anode Enabled by a Thiourea‐Derived Protective Layer for Alkaline Zinc Batteries
Source: Adv Sci (Weinh). 2026 Jun 4;13(48):e75929. doi: 10.1002/advs.75929 (PMC13336887; doi:10.1002/advs.75929)
Supplement: Supplementary file 1 [file ADVS-13-e75929-s001.pdf]

Supporting Information

Highly Reversible Zinc Anode Enabled by a Thiourea-Derived Protective Layer for Alkaline Zinc Batteries

Noam Navon,[1] Alagar Raja Kottaichamy,*[1,2,3] Thamaraichelvan Marichelvam,[1] Jonathan Tzadikov,[1] Michael Volokh,[1] Liel Abisdris,[1] Alexander Upcher,[4] and Menny Shalom*[1,4]

[1]Noam Navon, Dr. Alagar Raja Kottaichamy, Dr. Thamaraichelvan Marichelvam, Dr. Jonathan Tzadikov, Dr. Michael Volokh, Dr. Liel Abisdris, Prof. Menny Shalom
Department of Chemistry, Ben-Gurion University of the Negev
Beer-Sheva 814105, Israel.
E-mail: mennysh@bgu.ac.il

[2]Dr. Alagar Raja Kottaichamy
Academy of Scientific and Innovative Research (AcSIR), Ghaziabad 201002, India.

[3]Dr. Alagar Raja Kottaichamy
Electrochemical Power Sources Division, CSIR-CECRI, Karaikudi 630003, Tamil Nadu, India.
E-mail: alagarraja.cecri@csir.res.in

[4]Dr. Alexander Upcher, Prof. Menny Shalom
Ilse Katz Institute for Nanoscale Science and Technology, Ben-Gurion University of the Negev, Beer-Sheva 814105, Israel.

Experimental Section

1. Materials

All reagents, solvents, and materials were purchased from the indicated commercial manufacturers and used as received unless stated otherwise. Thiourea (thiocarbamide, TU, ≥ 99%) from Thermo Scientific; potassium hydroxide pellets (KOH, AR grade) from Macron Fine Chemicals; sulfur (powder, 99%), Nafion (5 wt% in lower aliphatic alcohols and water, contains 15–20% water), and hydrogen peroxide (H_2O_2, 30 wt%) from Sigma-Aldrich; urea (98%) from Glentham Chemicals; zinc acetate dihydrate ($Zn(OAc)_2 \cdot 2H_2O$, ACS grade) from Merck; ethanol (EtOH, technical grade 99%) from Bio-Lab, Israel; 2-propanol (IPA, HPLC & spectroscopy grade) from Loba Chemie, India; anionic exchange membrane (Fumasep FAS-30) and carbon paper (TGP-H-60, Toray) from Fuel Cell Store, USA; Zn foil (0.62 mm thick) from Alfa Aesar (now Thermo Scientific); deionized water (DI water, 18.2 mΩ cm resistivity at 25 °C, purified using a Merck Millipore Direct-Q3 system) was used for all aqueous solutions. Oxygen (≥99.6% purity) and argon (99.999% purity) gases were procured from Maxima Ltd., Israel.

2. Characterization Details

X-ray diffraction (XRD). XRD patterns were obtained using a PANalytical Empyrean diffractometer (equipped with an X'celerator position-sensitive detector) with a scanning time of ≈15 min for a 2θ range of 5–80° using Cu Kα radiation (λ = 1.54178 Å, 40 kV, 30 mA).

X–ray photoelectron spectroscopy (XPS). The chemical states of key elements were analyzed from XPS measurements conducted on an X-ray photoelectron spectrometer (ESCALAB-Xi+) in an ultrahigh vacuum (4×10^{-10} bar) apparatus with an Al Kα X-ray source and a monochromator. The X-ray beam size was 900 μm. Depth profiles were obtained after Ar ion-gun etching for ~150 s, with a sputtering rate of 0.153 nm s^{-1}. All binding energies in the XPS spectra were calibrated using the C 1s peak at 284.8 eV and a pass energy of 20.0 eV.

Scanning electron microscopy (SEM). The surface morphology was analyzed by SEM using an FEI Verios 460L high-resolution FEG-source microscope with a through-lens (TLD) or Everhart–Thornley (ETD) detectors at U_0 = 3.50 kV and a probe current of 25 pA.

Fourier-transform infrared spectroscopy (FTIR). The presence of functional groups was analyzed by FTIR spectroscopy using a Thermo Scientific Nicolet iS5 spectrometer (KBr window; diamond iD7 attenuated total reflectance (ATR) optical base). Each measurement consists of 16 scans at 4 cm^{-1} resolution in the 400–4000 cm^{-1} range.

Gas chromatography (GC). Hydrogen evolution was quantified by gas chromatography (Agilent 7820 GC system equipped with CP-Molecular Sieve 5A and PoraPLOT Q columns, coupled to a thermal conductivity detector, TCD).

Contact angle measurement. The contact angles between the bZn, hZn, and pZn_{50} electrodes and the electrolyte were measured three times for each sample using the OCA contact angle system (DataPhysics Instruments).

Focused ion beam (FIB). To prepare a cross-sectional sample of the ZnS/CN coating layer for scanning transmission electron microscopy, an FEI Helios G4 UC system (Thermo Fisher Scientific) was used, coupled to a gallium focused ion beam.

3D laser scanning confocal microscopy. The film thickness was measured by manually scratching the coated electrode with a needle to expose the underlying Zn substrate. Then, a LEXT OLS5000 was used to obtain the thickness profile under low magnification (10×) by focusing on a defined scan area around the scratch, and the film thickness was determined as the vertical distance between the lowest point corresponding to the exposed Zn substrate and the average height of the coating adjacent to the scratch.

Scanning/transmission electron microscopy (S/TEM). A Spectra 200 (Thermo Fisher Scientific) microscope (S/TEM), equipped with a probe Cs-corrector (S-CORR) and a cold-FEG electron source, at U_0 = 200 kV was used. In TEM mode, the images and the electron diffraction patterns were recorded with Ceta-M CMOS camera. In STEM mode, the images were recorded with a high-angle annular dark-field (HAADF) detector, a semi-convergence angle of 30 mrad, a typical probe current of 250 pA, and a collection angle range of 57.0–200.0 mrad. The energy dispersive X-ray spectroscopy (EDS) spectra were collected utilizing a Super-X (Thermo Fisher Scientific) four-segment silicon drift detector (SDD). The images and the EDS spectra were processed with Thermo Fisher Scientific Velox software (version 3.15).

3. Electrochemical Methods

For Zn||Zn symmetric cells, identical pairs of electrodes (bZn, hZn, and pZn_x, x = 20, 40, 50, 80 layers) were assembled into an SVC-3 Voltammetry cell (SVC-3, ALS Co., Japan), containing 15 mL of electrolyte (6 M KOH + 0.20 M $Zn(OAc)_2$). For long-term durability tests of a symmetric cell at 2 mA cm^{-2} and 10 mAh cm^{-2}, a larger cell containing 70 mL of the same electrolyte was used. Electrochemical measurements were performed using a two-electrode arrangement connected to a potentiostat, either an Ivium-n-Stat multichannel electrochemical analyzer (Ivium, Netherlands) or a Biologic VMP 300, with a carbon holder serving as a connector to the fabricated electrodes. Before each measurement, electrochemical cycling was performed in the corresponding electrolyte to clean the electrode surface.

The electrochemical performance of both symmetrical cells and complete battery systems was evaluated using chronopotentiometry. This involved subjecting the cells to galvanostatic charge–discharge protocols versus (vs) Zn^{2+}/Zn across a range of current densities (5–30 mA cm^{-2}) while recording potential variations as a function of time. This methodology provided a comprehensive assessment of cycling efficiency, rate capability, and long-term operational stability under various electrochemical conditions.

Hydrogen evolution analysis was performed in a two-compartment H-cell (C027-2, Xian Yima Optoelec Co., Ltd., China; each compartment 100 mL), separated by an anion-exchange membrane, with bZn, hZn, or pZn_{50} anodes and $Ni_{20}Py$ catalyst[1] air cathodes. Each half-cell contained 70 mL of electrolyte (6 M KOH + 0.20 M $Zn(OAc)_2$, anodic; 6 M KOH, cathodic). The evolved hydrogen was measured at regular ~15 min intervals during cycling (during charging) at 2 mA cm^{-2} and an areal capacity of 5 mAh cm^{-2}.

4. Materials Preparation

Preparation and cleaning of zinc substrates. For all zinc substrates, a preliminary cleaning procedure was used. The commercial zinc foil surface preparation involved manual removal of the native oxide layer using P320 sandpaper, followed by sequential cleaning in 1% m/v Alconox detergent solution (5–60 min), rinsing with ethanol, and 30 minutes of ultrasonication in IPA.

Preparation of pZn_x electrodes. A thiourea precursor solution (125 g L^{-1}) was prepared by dissolving 10 g of thiourea in 80 mL of deionized water and thoroughly mixing for 15 minutes until complete solubilization occurred. The prepared solution was then deposited onto zinc foil substrates (1.5 cm^2 for small electrodes; 5.0 cm^2 for large electrodes) maintained at 50 °C utilizing a Sono-Tek ExactaCoat ultrasonic spray system. The experimental parameters include varying the number of spray cycles on each side of the Zn foils (20, 40, 50, or 80), shaping the air pressure of the spray (0.5 psi), dwell time between spray cycles (10 s), and distance between the spray nozzle and the Zn electrode (4.0 cm). To this end, the Zn foils with sprayed thiourea layers are placed in a glass tube (16 mm in diameter × 100 mm in length). The pre-thermal treatment electrodes are noted as $Zn\text{-}TU_x$ (x = 20, 40, 50, or 80). The glass tube is covered with aluminum foil and heated to 350 °C in a tube furnace under N_2 atmosphere (99.999%) according to the following program:

1. From room temperature to 60 °C (initial heating); step duration: 1 min.
2. From 60 °C to 120 °C; step duration: 60 min (heating rate of 1 °C min^{-1}).
3. From 120 °C to 350 °C; step duration: 46 min (heating rate of 5 °C min^{-1}).
4. At the target temperature of 350 °C, the dwell time is 2 hours.
5. Cool down naturally from 350 °C to room temperature.

Preparation of hZn electrodes. Following the cleaning procedure, these electrodes were directly placed in 16 mm glass tubes, capped with aluminum foil, and processed in a tube furnace under a N_2 atmosphere (99.999%) using the same heating program as for the pZn electrodes.

Preparation of Zn@CN electrodes. An aqueous urea solution (375 g L^{-1}) was prepared by dissolving 30 g of urea in 80 mL of deionized water and thoroughly mixing

for 15 minutes until complete solubilization occurred. The prepared solution was then deposited onto zinc foil substrates (1.5 cm^2), maintained at 50 °C utilizing a Sono-Tek ExactaCoat ultrasonic spray system. The experimental parameters include the number of spray cycles (80), shaping air pressure of the spray (0.5 psi), dwell time between spray cycles (10 s), and distance between the spray nozzle and the Zn electrode (4.0 cm). To this end, the Zn foils with the sprayed urea layers are placed in a glass tube (16 mm diameter × 100 mm length) containing 0.5 g of urea powder at the bottom. The glass tube is covered with aluminum foil and heated to 350 °C in a tube furnace under N_2 atmosphere (99.999%) according to the following program:

1. From room temperature to 60 °C (initial heating); step duration: 1 min.
2. From 60 °C to 120 °C; step duration: 60 min (heating rate of 1 °C min^{-1}).
3. From 120 °C to 350 °C; step duration: 46 min (heating rate of 5 °C min^{-1}).
4. At the target temperature of 350 °C, the dwell time is 2 hours.
5. Cool down naturally from 350 °C to room temperature.

Preparation of Zn@ZnS electrodes. To prepare the Zn@ZnS electrodes, zinc foil substrates (1.5 cm^2) were placed in a glass tube (16 mm diameter × 100 mm length) containing 1.06 g of sulfur powder at the bottom. The glass tube is covered with aluminum foil and heated to 350 °C in a tube furnace under N_2 atmosphere (99.999%) according to the following program:

1. From room temperature to 60 °C (initial heating); step duration: 1 min.
2. From 60 °C to 120 °C; step duration: 60 min (heating rate of 1 °C min^{-1}).
3. From 120 °C to 350 °C; step duration: 46 min (heating rate of 5 °C min^{-1}).
4. At the target temperature of 350 °C, the dwell time is 2 hours.
5. Cool down naturally from 350 °C to room temperature.

5. Hydrogen Evolution Quantification

The evolved H_2 gas in the anodic compartment was quantified at regular time intervals using a gas chromatograph (Agilent 7820 GC system) during the Zn reversibility cycling process, performed at a current density of 2 mA cm^{-2} and an areal capacity of 5 mAh cm^{-2}. The measurements were conducted in a custom-designed two-compartment cell (each compartment of 100 mL volume) separated by an anion-exchange membrane (Fumasep FAS-30, Fuel Cell Store, USA), cut into ~3 cm^2. The cathodic compartment was filled with 6 M KOH aqueous solution, while the anodic compartment contained 6 M KOH with 0.20 M $Zn(OAc)_2$. To monitor hydrogen evolution, 200 µL gas samples were periodically extracted from the 30 mL headspace above the 70 mL electrolyte in the anodic compartment at ~15-minute intervals using a Pressure-Lok precision analytical syringe (A-2 Luer lock, Vici). Prior to each measurement, the electrolyte in the anodic compartment was purged with Ar gas for 20 minutes.

6. Rechargeable Zn–Peroxide Battery (RZPB) Assembly and Study

Preparation of zinc anodes. The pZn_{50} anode was prepared using the same coating method described earlier. For the bare Zn electrode, surface cleaning and mechanical polishing were performed as previously described.

Preparation of air cathodes. 70 mg of the Ni-based bifunctional catalyst ($Ni_{20}Py$)[1] was dispersed in 2 mL IPA with 70 µL of 5 wt% Nafion solution. The mixture was ultrasonicated for 3 h to form a homogeneous ink, which was then drop-cast onto carbon paper measuring 2 × 2.5 cm^2 and left to dry for approximately 1 h. The resulting areal catalyst loading was approximately 1 mg cm^{-2}. The rechargeable Zn–peroxide battery was tested in home-built electrochemical cells.[1] A two-electrode configuration was used by pairing $Ni_{20}Py$ loaded on carbon paper as the cathode electrode (area = 5 cm^2, catalyst loading 1 mg cm^{-2}) in 6 M KOH in the first compartment and a Zn foil anode (bZn or pZn_{50}) in 6 M KOH with 0.20 M $Zn(OAc)_2$ dissolved in the anodic compartment. Both compartments were filled with approximately 15 mL of electrolyte and separated by a Fumasep (FAS-30) anionic exchange membrane to facilitate ionic transport. During discharge and charge cycling, either high-purity oxygen (≥99.6%) or ambient air was continuously supplied to the cathode side to ensure oxygen saturation.

Calculation of round-trip energy efficiency (*η*) of rechargeable Zn–peroxide battery. The energy efficiency was calculated as $\eta = \frac{V_{\mathrm{discharge}}}{V_{\mathrm{charge}}} \times 100\%$

Supporting Information Figures

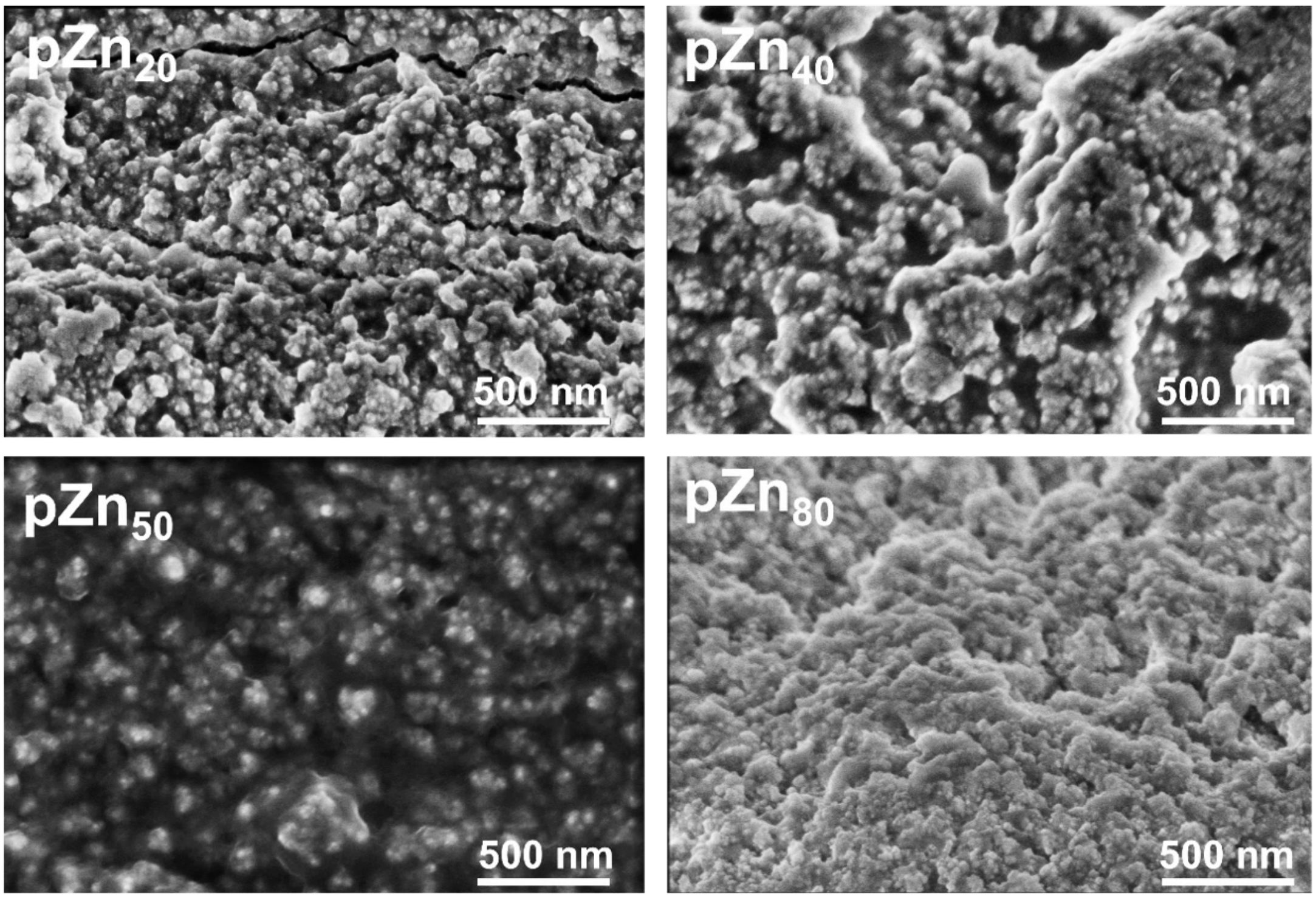

Figure S1. Top-view SEM images of pZn_{20}, pZn_{40}, pZn_{50}, and pZn_{80} electrodes.

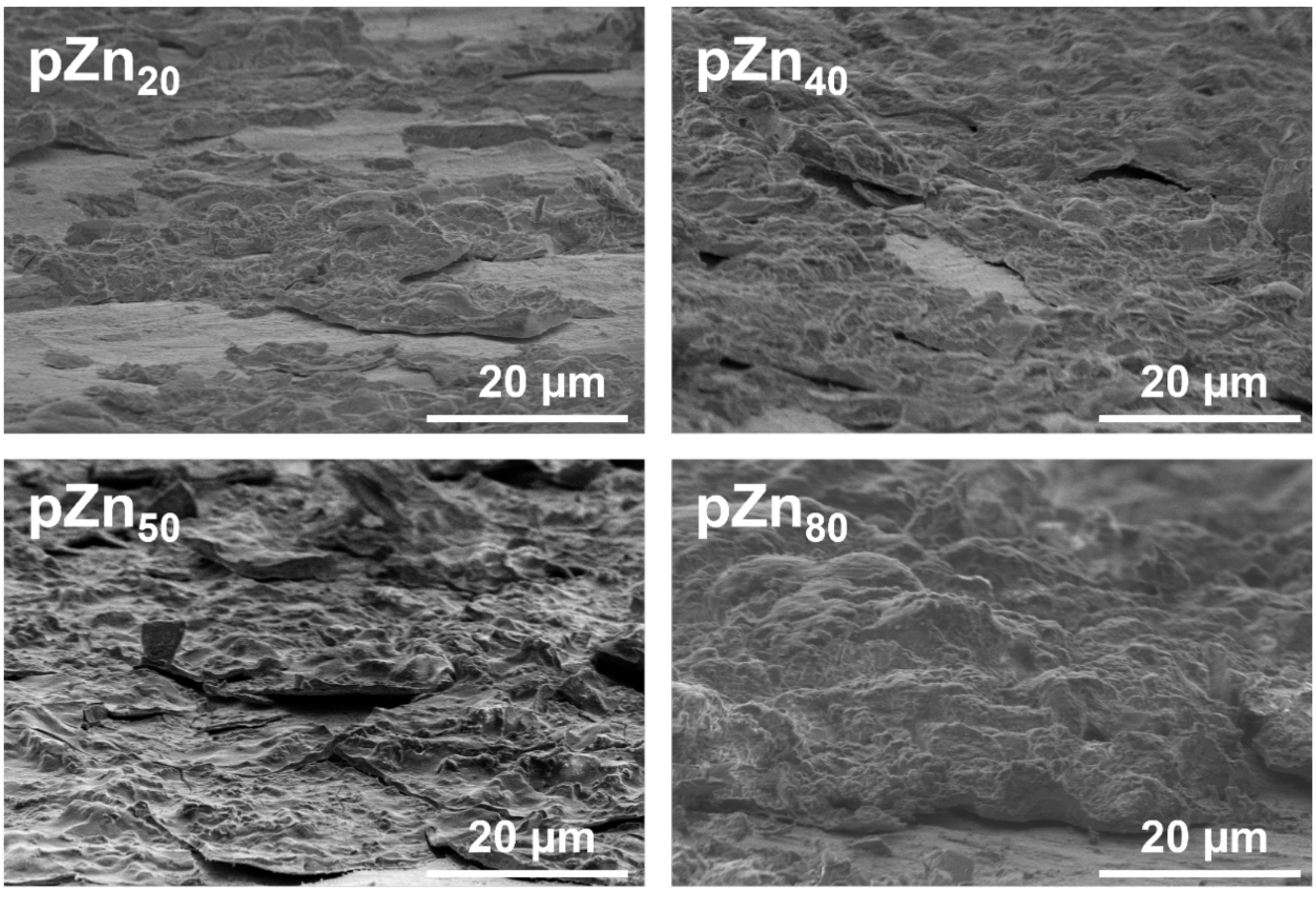

Figure S2. Tilted-view SEM images of pZn_{20}, pZn_{40}, pZn_{50}, and pZn_{80} electrodes.

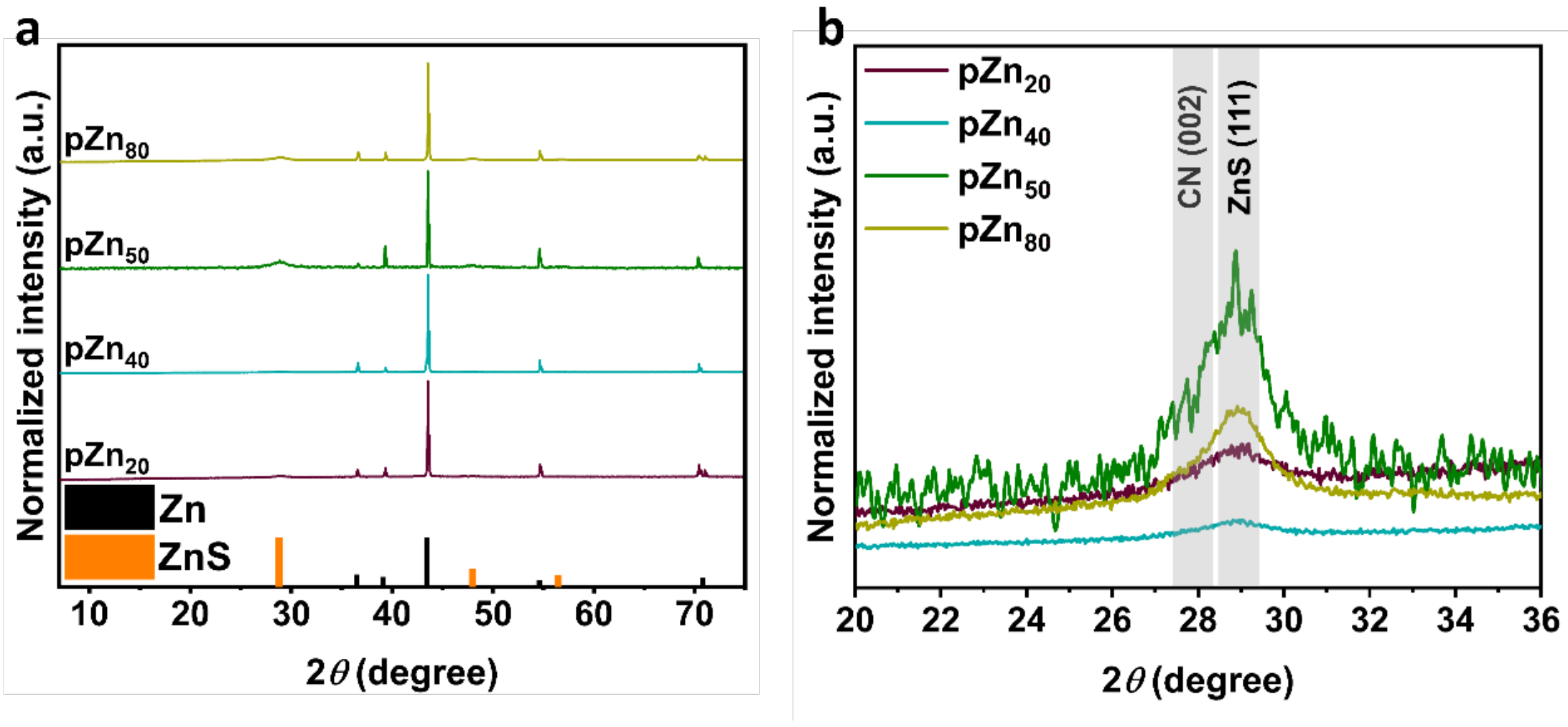

Figure S3. (a) XRD patterns of pZn_{20}, pZn_{40}, pZn_{50}, and pZn_{80} electrodes (the patterns are vertically offset for clarity) with literature (ICDD) diffraction stick patterns (Zn in black and ZnS in orange); each pattern in normalized to the Zn (101) diffraction intensity ca. 43.47°, (b) magnified view of the 2θ = 20–36° region showing the characteristic diffraction signals of cubic ZnS (111) and CN (equivalent to the spacing of graphitic C_3N_4's (002) interlayer stacking).

<u>*ICDD* cards</u>

00-065-0723 – cubic ZnS (space group F-43m)

00-001-1238 – hexagonal Zn (space group P63/mmc)

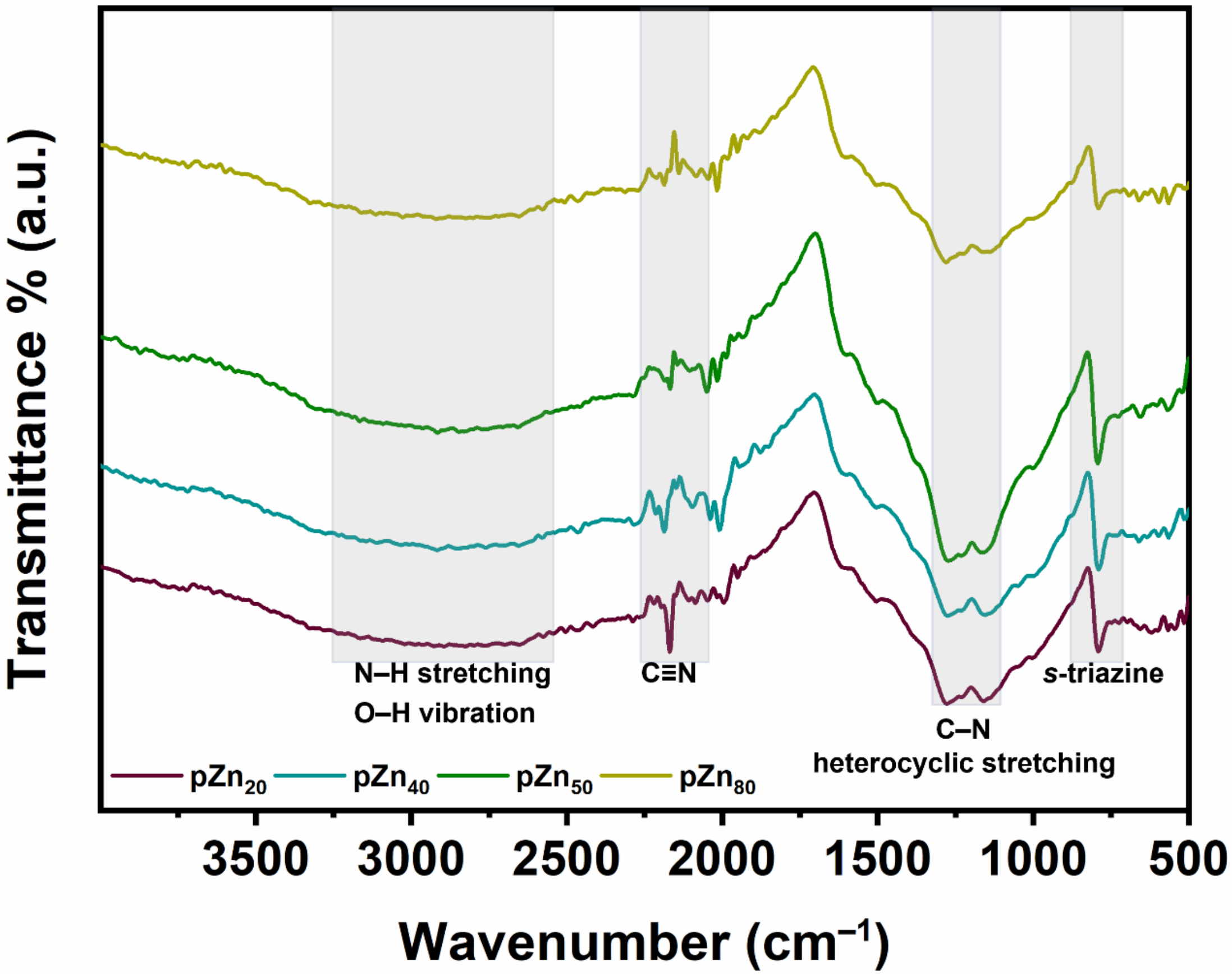

Figure S4. Fourier-transform infrared spectroscopy (FTIR) spectra of pZn_{20}, pZn_{40}, pZn_{50}, and pZn_{80} electrodes. The spectra are vertically offset for clarity.

FTIR measurements confirm the formation of a partially condensed polymeric carbon nitride layer. The peak at 792 cm^{-1} is associated with the vibration characteristics of *s*-triazine units; the absorption bands in the 1200–1500 cm^{-1} range correspond to C–N heterocyclic stretching modes; the peak at 2167 cm^{-1} indicates the presence of cyano group (C≡N); and the broad peak at 3000–3500 cm^{-1} is associated with N–H stretching vibration from uncondensed amino group along with O–H vibration from adsorbed water molecules.[2–4]

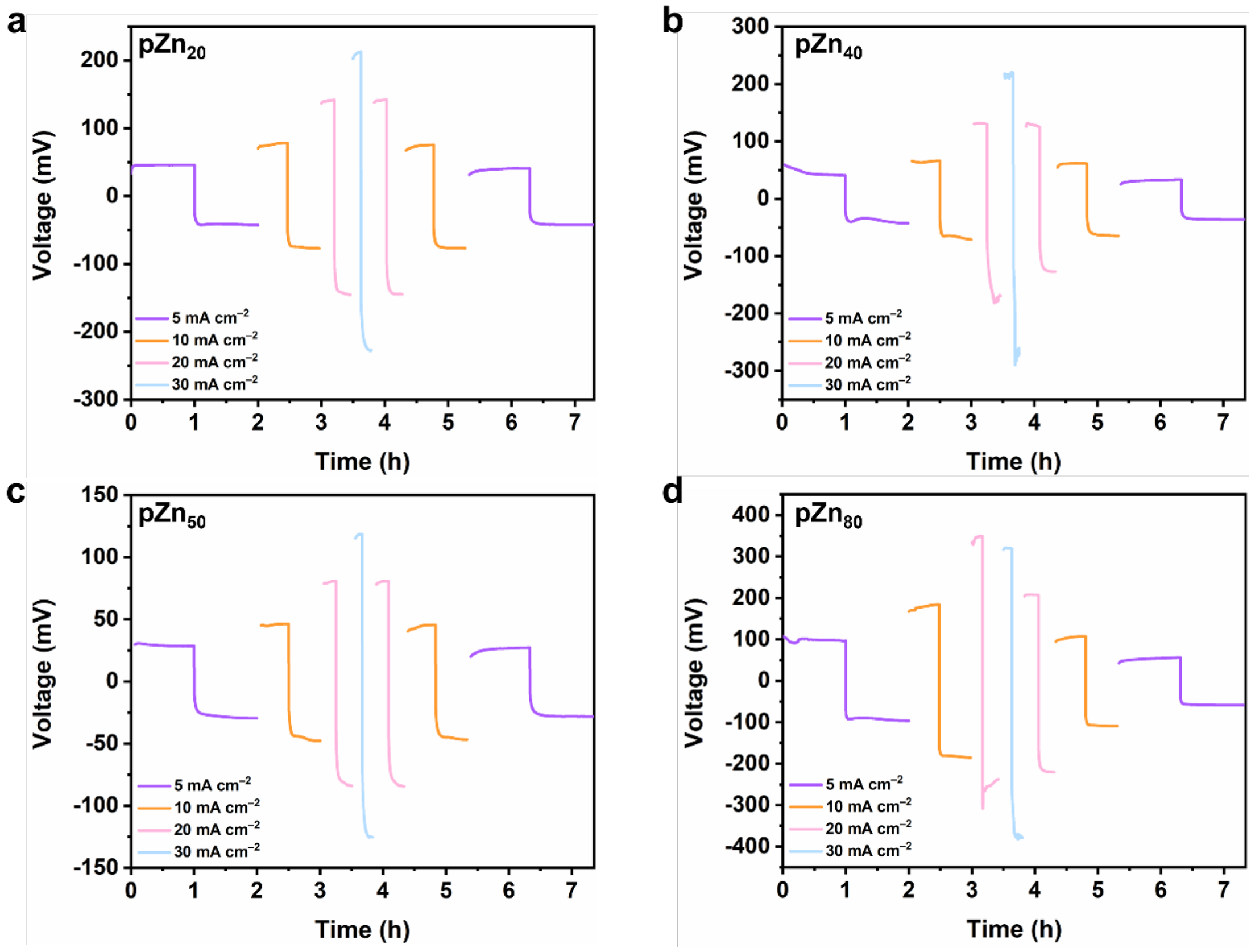

Figure S5. Rate profiles for Zn||Zn symmetric cells at a current density of 5–30 mA cm^{-2} with a capacity of 5 mAh cm^{-2} with different layers: (a) 20 layers, (b) 40 layers, (c) 50 layers, and (d) 80 layers.

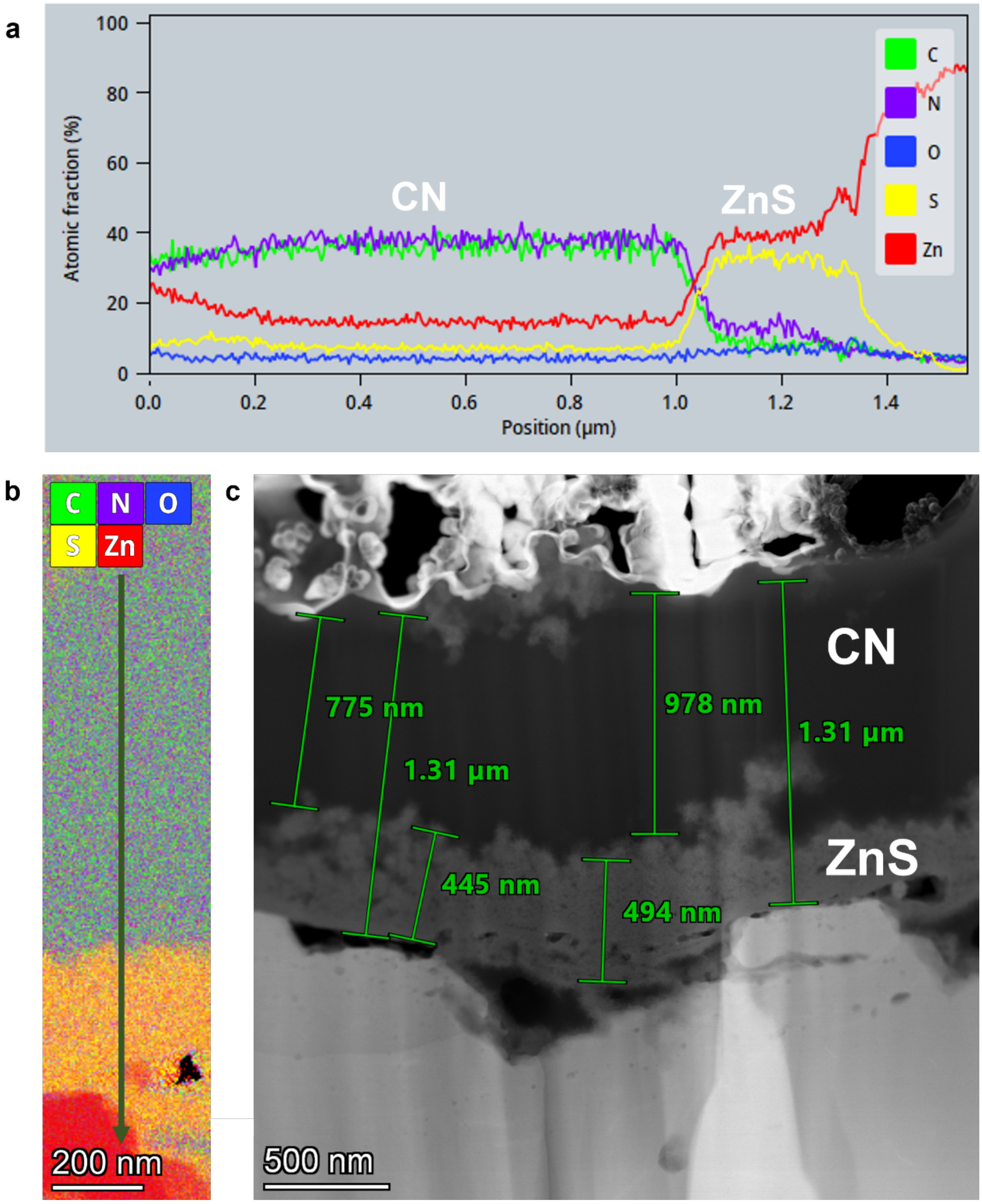

Figure S6. Cross-sectional STEM–EDS analysis of pZn_{50}: (a) Atomic fraction profile of the elements along the cross section, (b) elemental mapping with the profile scan direction indicated by the arrow, (c) a layer thickness measurement.

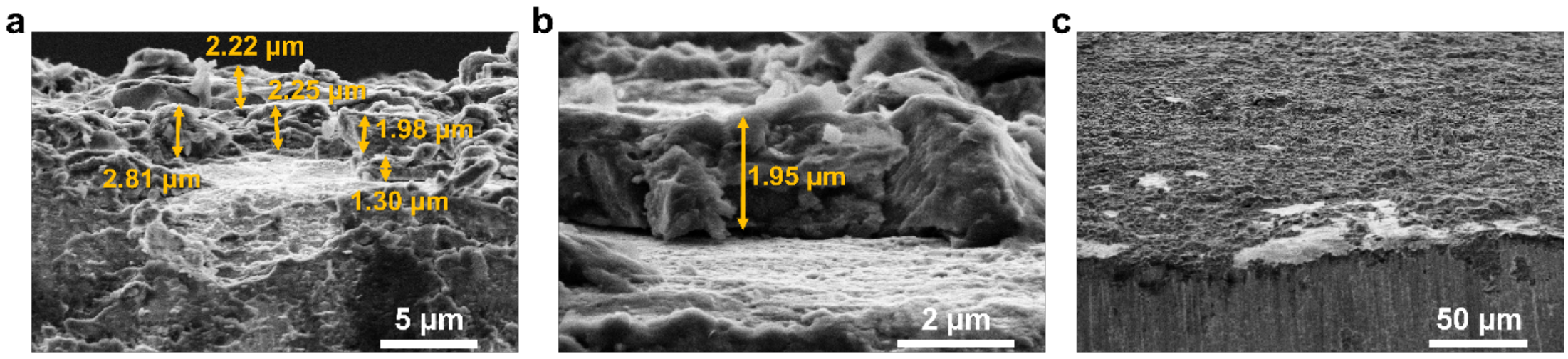

Figure S7. (a,b) Cross-sectional SEM images of a pZn_{50} electrode taken from two different regions and (c) tilted-view SEM image of a pZn_{50} electrode at a 20° tilt angle.

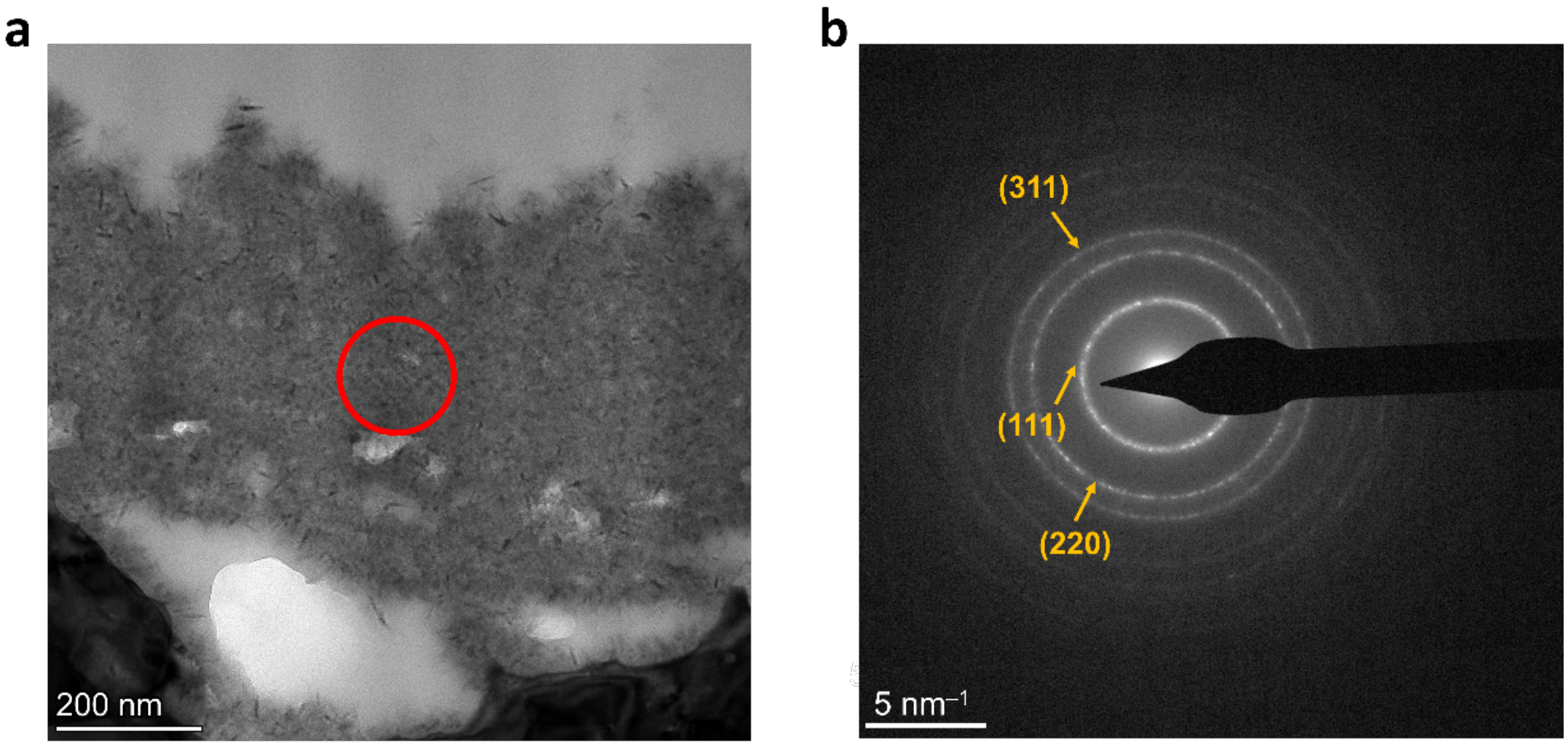

Figure S8. (a) TEM image of the pZn_{50} coating cross-section; the position of the selected area aperture for SAED is indicated by the red circle, (b) SAED pattern with diffraction rings corresponding to (111), (220), and (311) plans, confirming the formation of ZnS.[5]

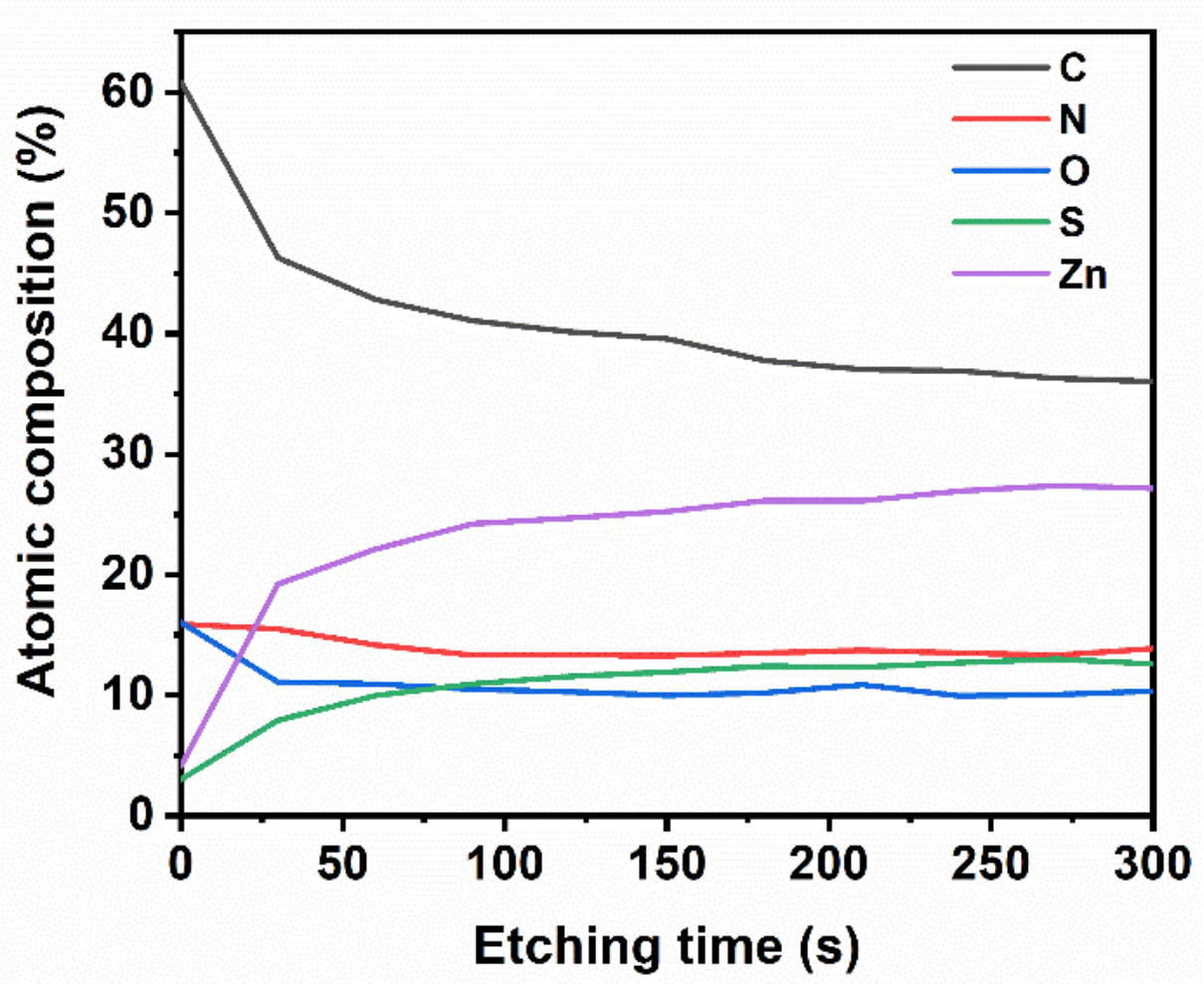

Figure S9. Compositional XPS depth profiles of pZn_{50} showing the C, N, O, S, and Zn percentages as a function of etching time.

The XPS depth-profiling results confirm the presence of both the CN top layer and ZnS species within the near-surface region of the protective coating, consistent with the STEM–EDS analysis (Figure S6). During the initial etching period (0–50 s), high carbon (~60%) and nitrogen (~16%) atomic compositions are observed, corresponding to the CN top layer. As etching progresses beyond 50 s, a gradual increase in Zn and S atomic fractions is observed, indicative of ZnS species within the CN top layer.

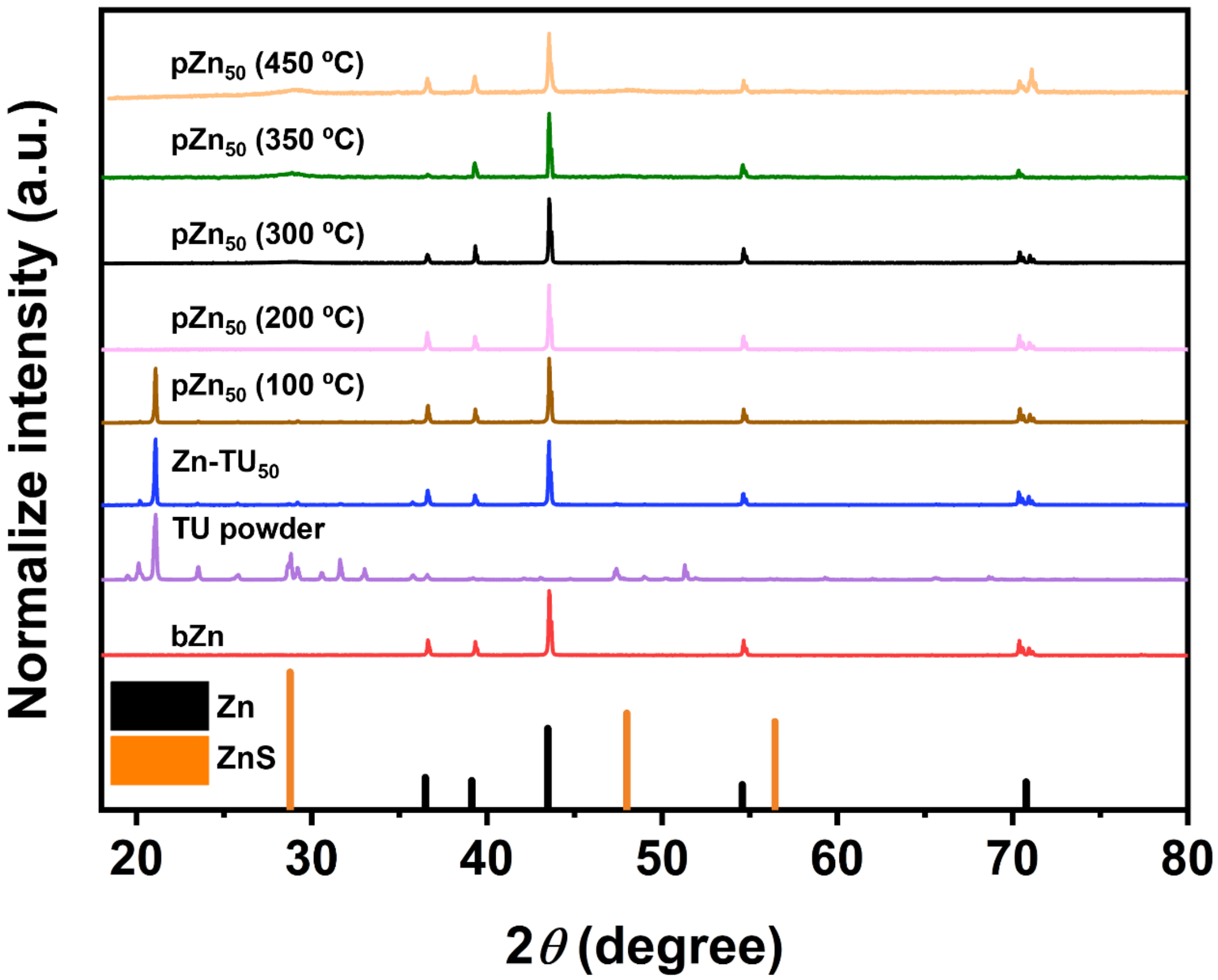

Figure S10. XRD patterns of bZn, thiourea precursor powder (TU), the resulting coated Zn sheets (Zn-TU_{50}), and pZn_{50} that was thermally treated at various temperatures (100, 200, 300, 350, and 450 °C). All patterns are vertically offset for clarity. All patterns from zinc-containing substrates are normalized to Zn's (101) diffraction ca. 43.47°. The TU powder pattern is normalized to its strongest diffraction ca. 21.1°.

A temperature-dependent XRD study was conducted to elucidate the mechanism of the protective layer formation. Zn foils coated with 50 layers of thiourea (Zn-TU_{50}) were subjected to thermal treatment at various temperatures (100, 200, 300, 350, and 450 °C). The successful thiourea deposition on Zn foil, as evidenced by the Zn-TU_{50} XRD pattern, served as the starting point for thermal treatment studies. Upon thermal treatment at 100 °C, the diffraction peaks of thiourea (i.e., the precursor) remain clearly visible, indicating that no thermal decomposition has occurred at this temperature, since the initial decomposition of thiourea takes place in the 140–180 °C temperature range.[6] Upon increasing the annealing temperature to 200 °C, the characteristic thiourea peaks disappear entirely, indicating initial decomposition of thiourea, and only bZn diffraction peaks remain. However, no additional peaks

corresponding to new phases were observed, suggesting that while thiourea decomposition has initiated, the intermediate species have not yet reacted with the zinc substrate to produce the ZnS/CN coating layer. When the annealing temperature is further increased to 300 °C, initial peaks corresponding to cubic ZnS emerge at ~28.8° and ~47.9°, confirming the onset of ZnS formation. Upon increasing the temperature to 350 °C, the intensity of the cubic ZnS diffraction peaks increases significantly, indicating enhanced ZnS crystallinity and a more complete protective layer formation, along with partial CN polymerization, as indicated by the broad peak at ~27.6°. In contrast, annealing at 450 °C, which exceeds the melting point of Zn, resulted in significant substrate deformation. Although the ZnS diffraction peaks are still observed, the Zn foil underwent partial melting and warping, rendering the electrode unusable for electrochemical applications. Therefore, 350 °C was determined as the optimal annealing temperature, providing sufficient thermal treatment for ZnS/CN formation while maintaining the structural integrity of the Zn substrate.

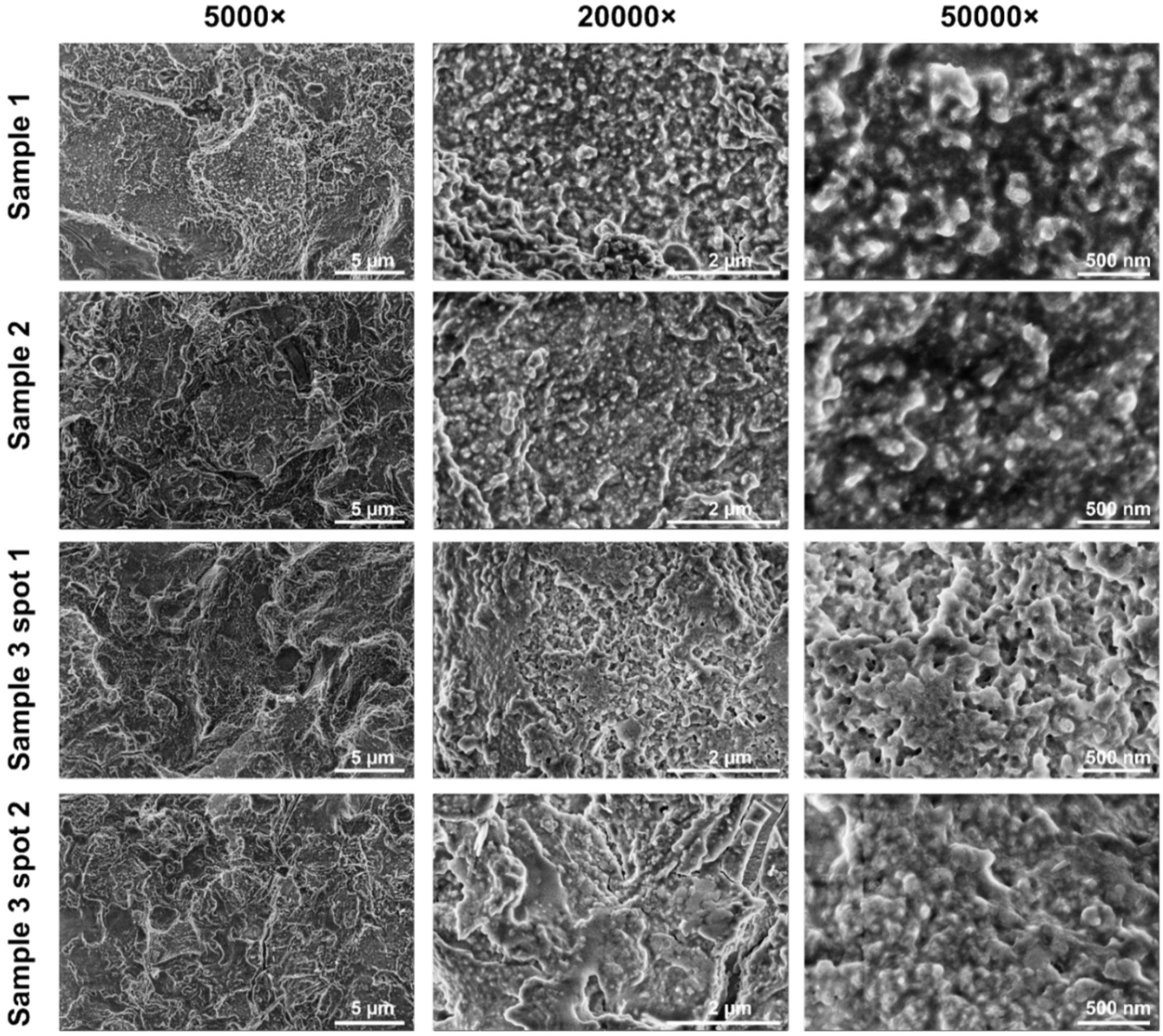

Figure S11. Top-view SEM images of three independently prepared pZn_{50} electrodes at 5000×, 20,000×, and 50,000× magnifications, demonstrating the uniformity and reproducibility of the ZnS/CN coating.

The uniformity and reproducibility of the protective layer were evaluated by scanning electron microscopy (SEM) analysis of three independently prepared pZn_{50} electrodes at different magnifications (5000×, 20,000×, and 50,000×). All three samples exhibit a highly consistent surface morphology, homogeneous coating coverage, and similar structural features, consistent with the top-view SEM images presented in Figure 3d and Figure S1. Furthermore, to assess spatial uniformity, we present SEM images from two distinct regions on sample 3. Both regions display identical morphological characteristics, consistent with those observed in the other two samples, confirming homogeneous ZnS/CN coverage across the entire electrode surface. These findings establish both the uniformity and reproducibility of the ZnS/CN protective layer across independently prepared samples, achieved through precise control of

processing parameters, including spray protocol, number of coating layers, and thermal treatment conditions.

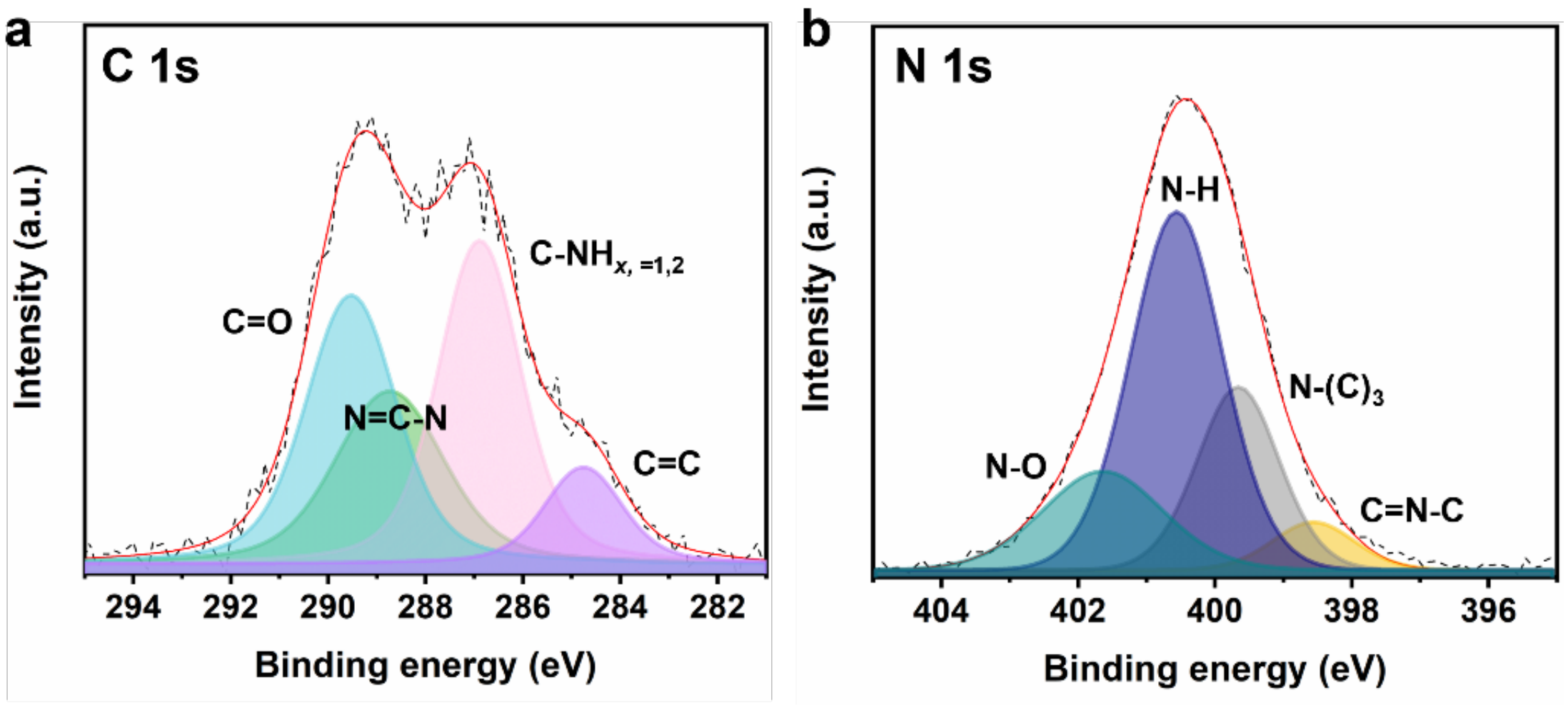

Figure S12. XPS spectra of Zn@CN: (a) C 1s, and (b) N 1s.

The C 1s spectrum shows four peaks at 284.8, 286.9, 288.7, and 289.5 eV, corresponding to C=C, C–NH$_x$ ($x = 1, 2$), N=C–N, and C=O, respectively.[7–9] The latter peak likely originates from partial surface oxidation during thermal treatment. The N 1s spectrum includes four peaks at 398.6, 399.7, 400.6, and 401.6 eV, assigned to C=N–C, N–(C)$_3$, N–H, and N–O species, respectively, with the N–O peak attributed to partial oxidation during thermal treatment.[10–14]

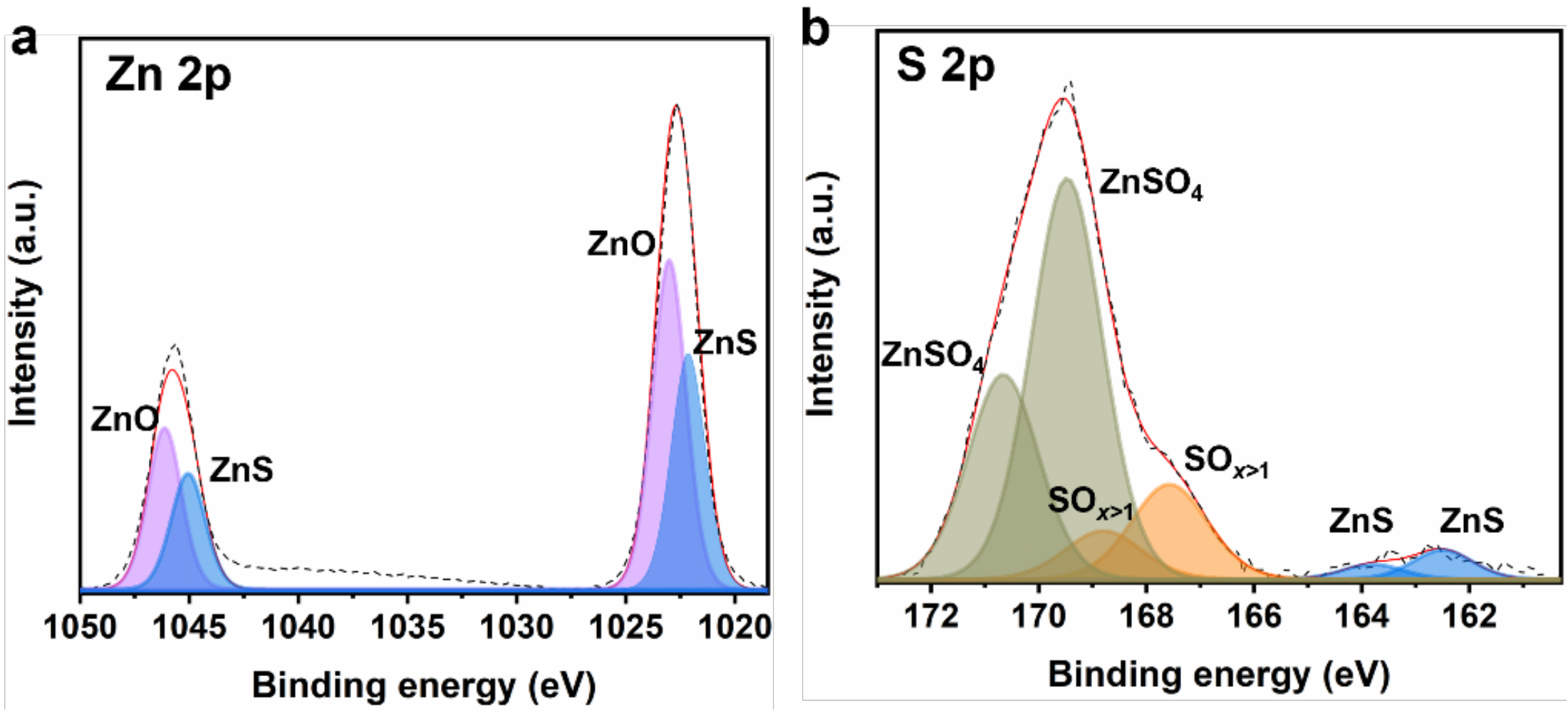

Figure S13. XPS spectra of Zn@ZnS: (a) Zn 2p, and (b) S 2p.

The Zn 2p spectrum exhibits two peaks at 1022.1 and 1045.1 eV, corresponding to the Zn $2p_{3/2}$ and Zn $2p_{1/2}$ transitions of ZnS, along with ZnO-related peaks at 1023.0 and 1046.1 eV, which are likely originating from surface oxidation of the zinc substrate during thermal treatment.[15,16] The S 2p spectrum displays two peaks at 162.5 eV (S $2p_{3/2}$) and 163.8 eV (S $2p_{1/2}$), corresponding to the S^{2-} species, confirming the formation of ZnS.[17] Additional peaks at 167.6 and 168.8 eV, assigned to S $2p_{3/2}$ and S $2p_{1/2}$ of SO_x ($x > 1$) species, indicate partial oxidation of the sulfidic components.[18,19] The peaks at 169.5 and 170.7 eV correspond to the S $2p_{3/2}$ and S $2p_{1/2}$ transitions of $ZnSO_4$, respectively.[20]

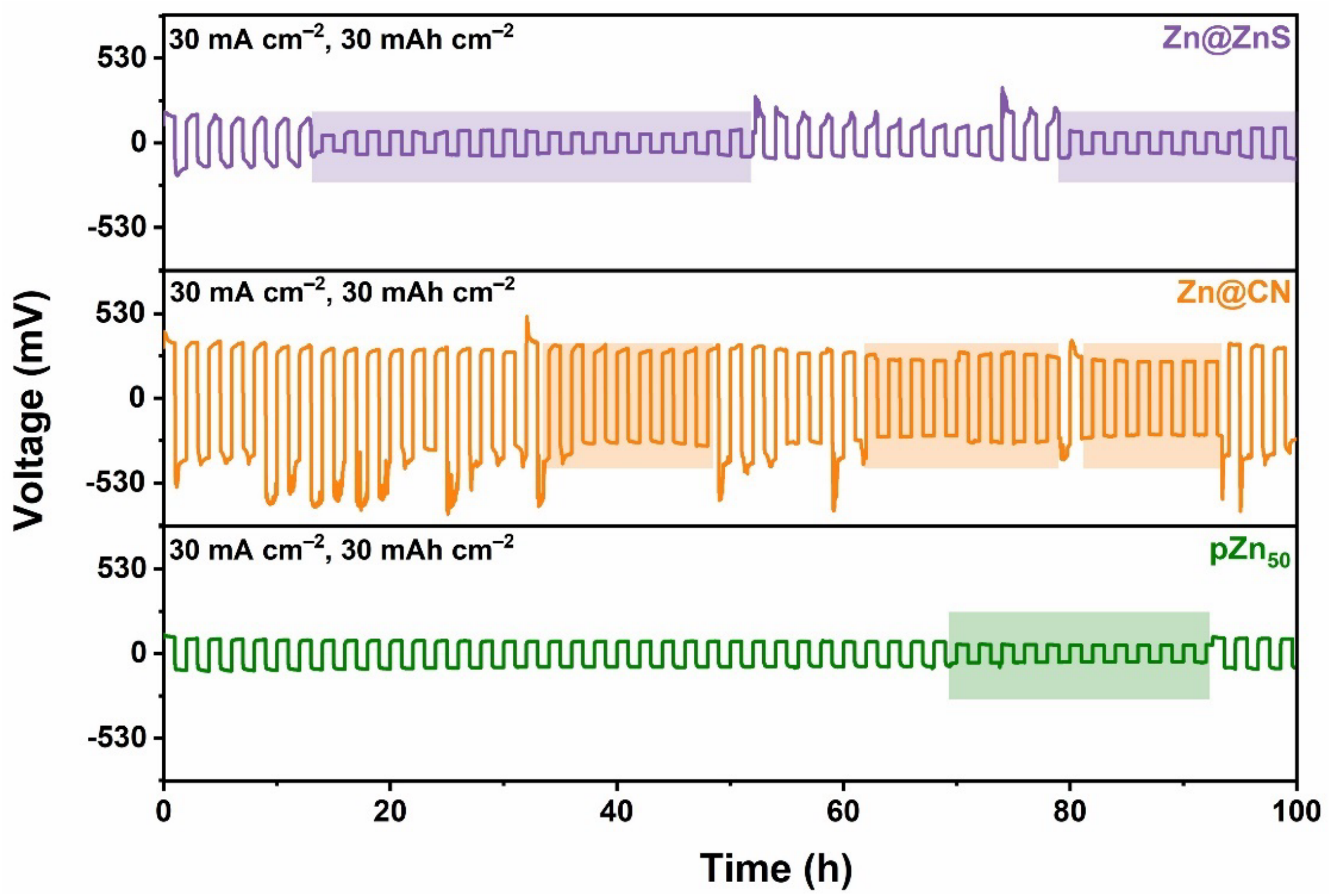

Figure S14. long-term cycling performance of Zn plating/stripping for pZn_{50}, Zn@CN, and Zn@ZnS electrodes at a high current density of 30 mA cm^{-2} with a fixed areal capacity of 30 mAh cm^{-2} in symmetric Zn||Zn cells. Colored markers indicate voltage drops due to dendrite contact with the opposing electrode (purple: Zn@ZnS; orange: Zn@CN; green: pZn_{50}).

To investigate the synergistic effect of the conjoint ZnS/CN structure, control experiments were performed using electrodes coated with either ZnS (Zn@ZnS) or CN (Zn@CN), and tested under identical symmetric cell conditions at a high current density of 30 mA cm^{-2} and a fixed areal capacity of 30 mAh cm^{-2}, as those presented for pZn_{50} in Figure 2d. In contrast to pZn_{50}, which exhibits a stable voltage profile with minimal fluctuations throughout the measurement, both Zn@ZnS and Zn@CN display pronounced voltage instabilities and significantly higher voltage hysteresis. Notably, the Zn@ZnS and Zn@CN electrodes exhibited multiple electrical disturbances throughout the measurement, as evidenced by voltage drops (background marked in purple for Zn@ZnS and in orange for Zn@CN) as zinc dendrites grow and reach the opposing electrode. These disturbances initiate at ~14 h for Zn@ZnS and ~30 h for Zn@CN, compared to pZn_{50}, which shows only a single voltage drop (marked in green) after ~70 h and maintains stable operation thereafter. These results reveal that dendrite growth is significantly faster and more extensive when only ZnS or CN is present as a

protective layer on the zinc anode. The synergistic combination of the two components in the ZnS/CN layer is essential for maintaining uniform Zn stripping/plating and effective dendrite suppression during high-capacity cycling.

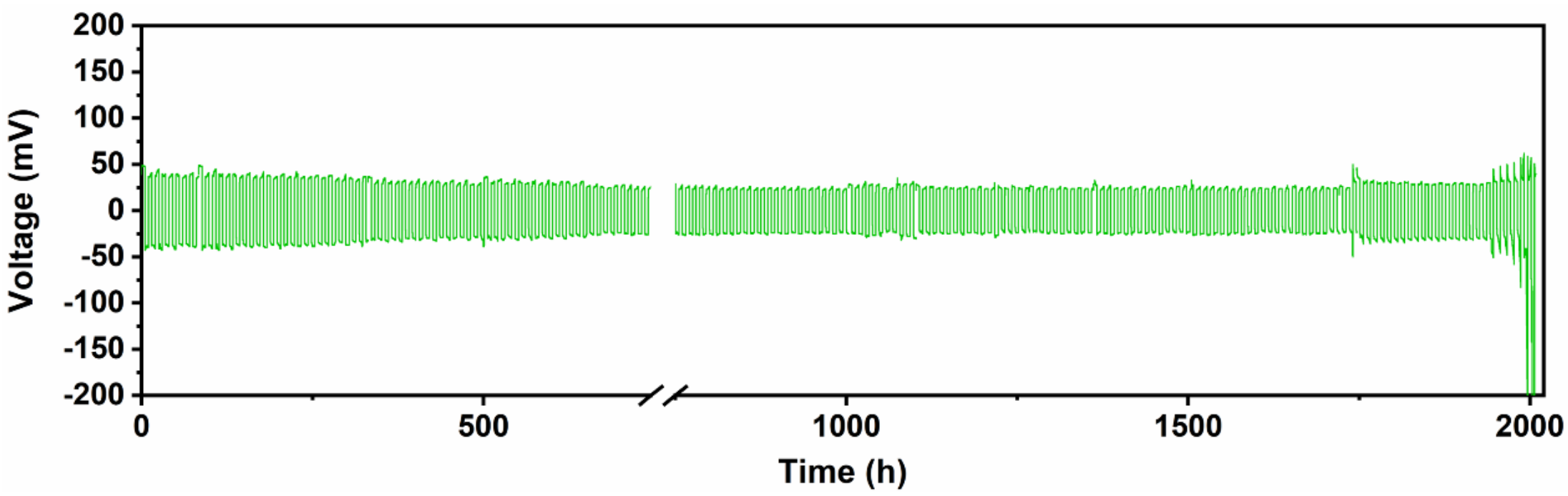

Figure S15. Long-term cycling performance of pZn_{50} plating/stripping at a current density of 2 mA cm^{-2} with a fixed areal capacity of 10 mAh cm^{-2} in symmetric Zn||Zn cells.

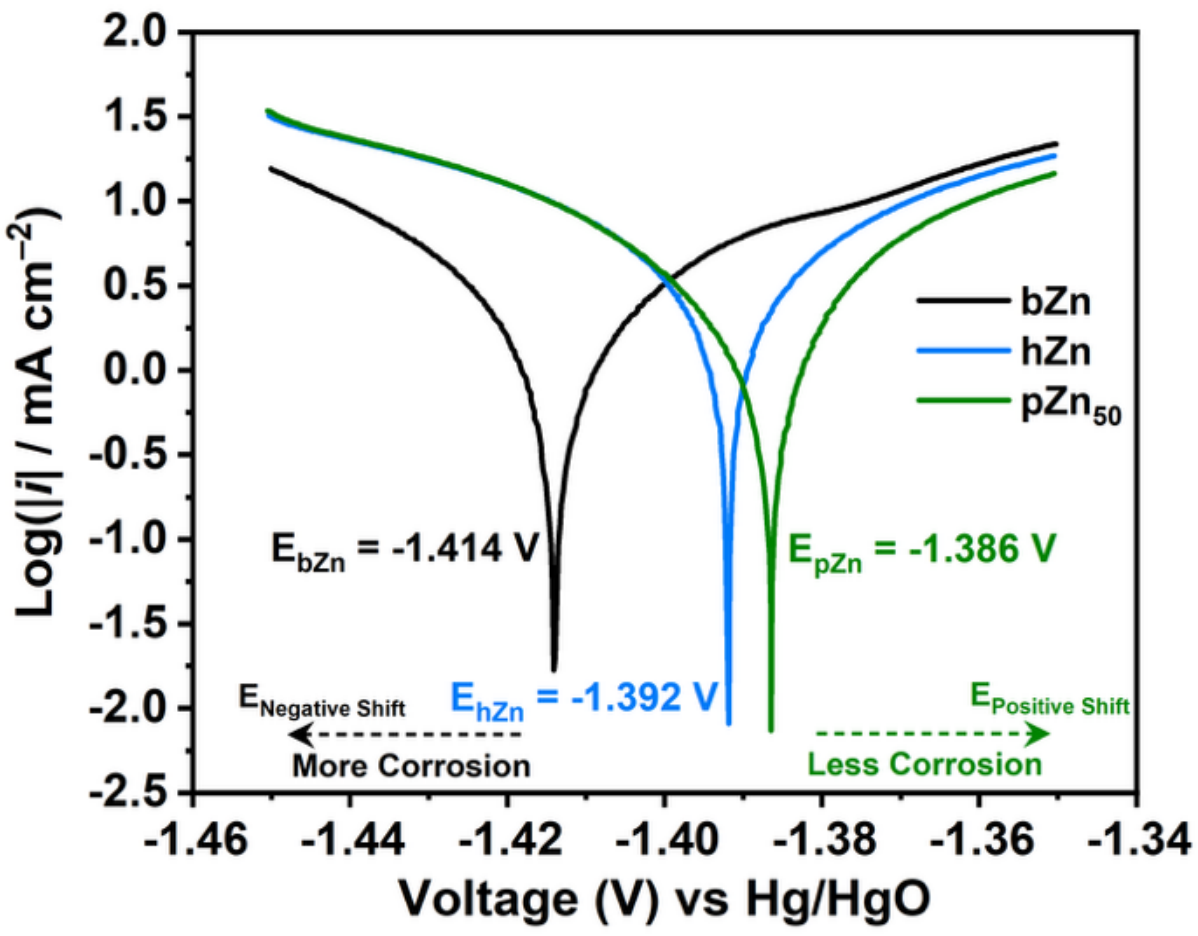

Figure S16. Tafel polarization plots for bZn, hZn, and pZn_{50} electrodes measured in a highly alkaline electrolyte (6 M KOH + 0.20 $Zn(OAc)_2$).

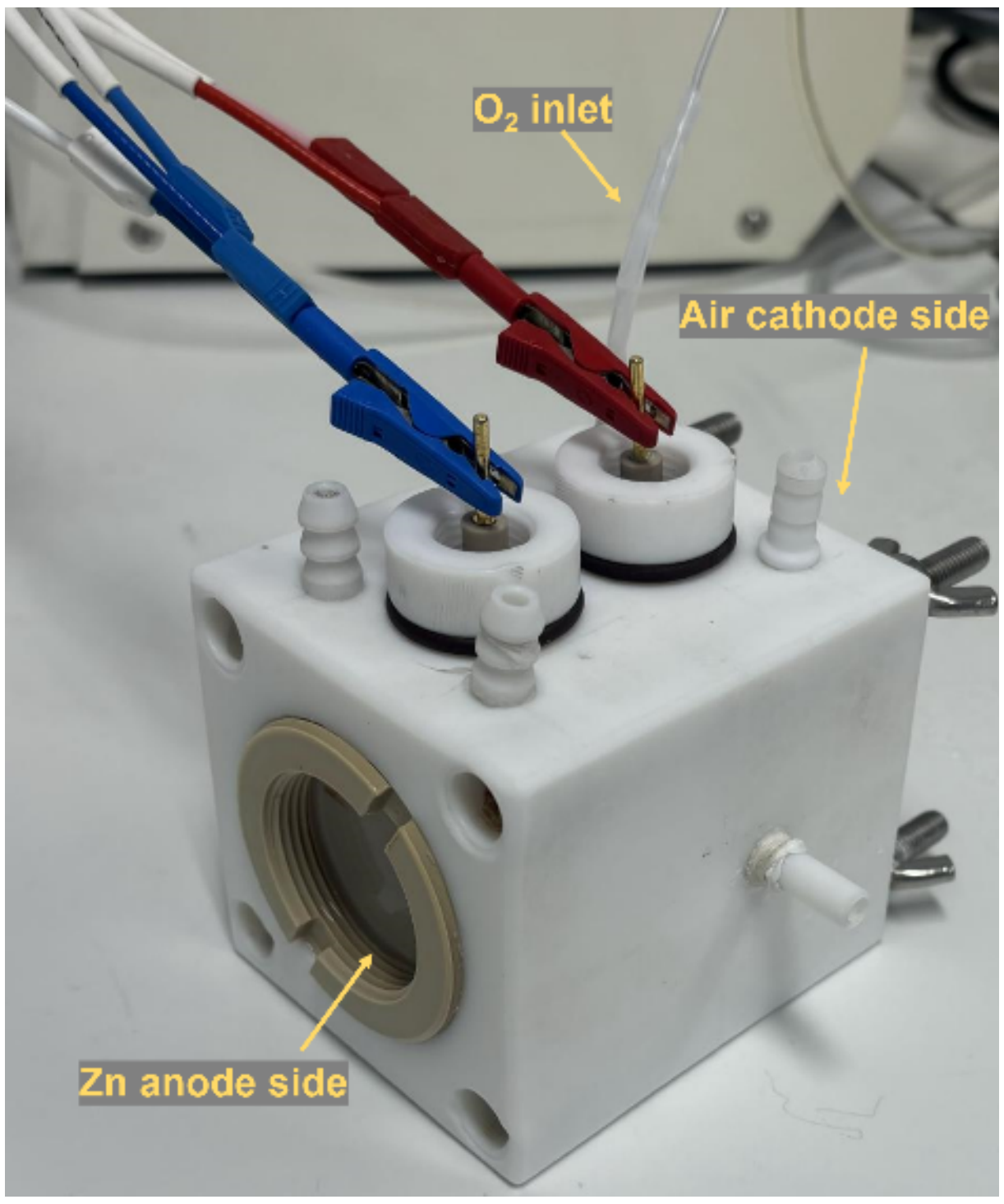

Figure S17. Two-compartment zinc–peroxide battery (ZPB) device.

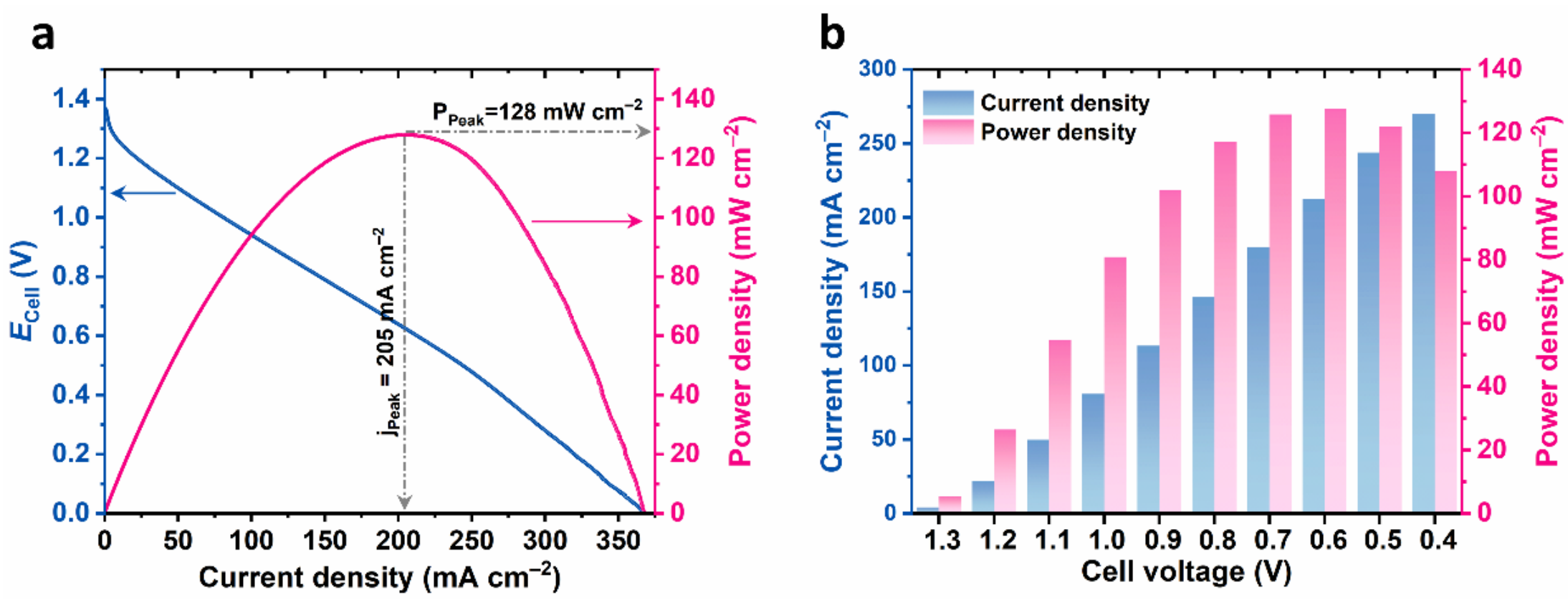

Figure S18. (a) Galvanodynamic polarization profile of ZPB and (b) the corresponding current densities and power densities at different cell potentials.

Supporting Information Tables

Table S1. Coating thickness of pZn_x electrodes, measured by 3D laser scanning confocal microscopy.

No.	Electrode	Film thickness (µm)
**1**	pZn_{20}	1.25
**2**	pZn_{40}	1.97
**3**	pZn_{50}	2.18
**4**	pZn_{80}	3.85

Table S2. Comparison of the electrochemical performance of zinc metal anodes reported in the literature in Zn||Zn symmetric cells at high current densities and areal capacities.

No.	Anode	Aqueous electrolyte	Current density (mA cm^{-2}) @ Capacity (mAh cm^{-2})	Number of cycles	Lifespan (h)	Ref.
**1**	**CN@Zn/ZnS**	**6 M KOH + 0.2 M $Zn(OAc)_2$**	**30@30**	**85**	**170**	**Present work**
**2**	IHS/Zn	3 M $ZnSO_4$	20@20	40	80	[21]
**3**	PA-Zn	2 M $ZnSO_4$	10@10	75	150	[22]
**4**	IAZO (ZnO layer on Zn anode)	2 M $ZnSO_4$	20@30	~26.7	~80	[23]
**5**	Zn (002)	$ZnSO_4$/DMA	30@30	60	120	[24]
**6**	Zn-002	2 M $ZnSO_4$	10@10	50	100	[25]
**7**	3D MXene array@Zn	3 M $Zn(CF_3SO_3)_2$	20@10	90	90	[26]
**8**	Zn@ZPO	3 M $Zn(CF_3SO_3)_2$	20@10	160	160	[27]
**9**	Zn@ZnP	2 M $ZnSO_4$	10@10<br>20@10	47.5<br>100	95<br>100	[28]

Table S3. Comparison of recently reported Zn||Zn symmetric cells with protective coatings on the Zn anode.

No.	Anode	Aqueous electrolyte	Current density (mA cm^{-2}) @ Capacity (mAh cm^{-2})	Number of cycles	Lifespan (h)	Voltage hysteresis (mV)	Ref.
1	ZnS/CN@Zn	6 M KOH + 0.2 M $Zn(OAc)_2$	2@10<br>30@30	1000<br>85	2000<br>170	49<br>170	Present work
2	CBL@Zn	6 M KOH	0.5@0.5	~100	~200	30	[29]
3	$Ti_3C_2T_X$ MXene/Zn	6 M KOH + 0.2 M $Zn(OAc)_2$	1@0.5	100	100	—	[30]
4	TiO_2@Zn	3 M $Zn(SO_3CF_3)_2$	1@1	75	150	57.2	[31]
5	Zn@UIO-66/rGx	2 M $ZnSO_4$	1@1	550	1100	26.5	[32]
6	TpBD-2F@Zn	2 M $ZnSO_4$	2@2	~600	~1200	24.4	[33]
7	N-C/Zn	2 M $ZnSO_4$	1@1	500	1000	64.1	[34]
8	Zn@ZnS	2 M $ZnSO_4$	0.5@0.25	300	300	42	[35]
9	Aramid@Zn	2 M $ZnSO_4$	1@1	~500	~1000	—	[36]
10	PVDF-Sn@Zn	2 M $ZnSO_4$	1@1	600	1200	—	[37]
11	ZnOHF NWs@Zn	2 M $ZnSO_4$	1@1	~350	~700	27.2	[38]
12	Nano-$CaCO_3$-coated Zn foil	3 M $ZnSO_4$ + 0.1 M $MnSO_4$	0.25@0.05	836	836	80–105	[39]
13	NGO@Zn	2 M $ZnSO_4$	1@1	600	1200	17	[40]
14	Al_2O_3@Zn	3 M $Zn(SO_3CF_3)_2$	1@1	250	500	36.5	[41]
15	Zn@PDA	2 M $ZnSO_4$ + 0.1 M $MnSO_4$	2@1	500	500	42	[42]
16	PSN Zn	2 M $ZnSO_4$	0.25@0.25	400	800	—	[43]
17	Sep-OH@Zn	2 M $ZnSO_4$	1@0.5	1000	1000	—	[44]
18	PTA@Zn	2 M $ZnSO_4$	1@1	600	1200	—	[45]
19	ZVO@Zn	2 M $ZnSO_4$ + 0.1 M $MnSO_4$	2@1	1000	1000	—	[46]
20	Zn@ZnF_2	2 M $ZnSO_4$	1@1	400	800	71.5	[47]

Table S4. Contact angle values of bZn, hZn, and pZn_{50} with 6 M KOH + 0.20 $Zn(OAc)_2$ electrolyte.

Sample name	Average (± standard deviation)
**bZn**	79.9° ± 1.3°
**hZn**	84.6° ± 4.9°
**pZn_{50}**	44.7° ± 3.3°

Table S5. Comparison of recently reported Zn–air batteries with protective coatings on the Zn anode.

No.	Anode	Cathode	Aqueous electrolyte	Current density ($mA\ cm^{-2}$)	Capacity ($mAh\ cm^{-2}$)	Lifespan (h)	Ref.
**1**	**CN@Z/ZnSn**	**$Ni_{20}Py$ loaded on carbon paper**	**6 M KOH / 6 M KOH + 0.2 M $Zn(OAc)_2$**	**2**	**9**	**1100**	**Present work**
**2**	Zn@CBL	20% Pt/C + RuO_2 catalyst	6 M KOH + 0.2 M $Zn(OAc)_2$	1<br>2	1<br>20	450<br>200	[29]
**3**	TAM-Tp-covered Zn	Co_3O_4 ink sprayed on a carbon cloth	6 M KOH + 0.2 M $Zn(OAc)_2$	10	5	600	[48]
**4**	Zn@PDA-UV	Pt/C-RuO_2 coated over the gas diffusion layer	6 M KOH + 0.2 M $Zn(OAc)_2$	5	0.42	72	[49]
**5**	ZnO-N-C-600	commercial cathodic catalyst (20% Pt/C+RuO_2)	6 M KOH + 0.2 M $Zn(OAc)_2$	5	—	300	[50]
**6**	PANI@ZnPc	Teflon layer, nickel mesh layer - carbon catalyst layer	7 M KOH	10	—	120	[51]

Supporting Information References

[1] A. R. Kottaichamy, J. Tzadikov, A. Pedersen, J. Barrio, G. Mark, I. Liberman, A. Upcher, M. Volokh, I. Hod, S. Barzilai, M. Noked, M. Shalom, "A Rechargeable Zn–Air Battery with High Energy Efficiency Enabled by a Hydrogen Peroxide Bifunctional Catalyst" *Adv Energy Mater* **2024**, *14*, 2403817.

[2] C. Tian, H. Zhao, J. Mei, S. Yang, "Cost-Efficient Graphitic Carbon Nitride as an Effective Photocatalyst for Antibiotic Degradation: An Insight into the Effects of Different Precursors and Coexisting Ions, and Photocatalytic Mechanism" *Chem. – Asian J.* **2019**, *14*, 162–169.

[3] H. Li, Y. Jing, X. Ma, T. Liu, L. Yang, B. Liu, S. Yin, Y. Wei, Y. Wang, "Construction of a well-dispersed Ag/graphene-like g-C_3N_4photocatalyst and enhanced visible light photocatalytic activity" *RSC Adv.* **2017**, *7*, 8688–8693.

[4] H. Deng, Y. Jia, W. Wang, S. Zhong, R. Hao, L. Fan, X. Liu, "Defect and Crystallinity-Mediated Charge Separation in Carbon Nitride for Synergistically Boosted Solar-Driven Hydrogen Evolution" *ACS Sustain. Chem. Eng.* **2023**, *11*, 13736–13746.

[5] P. Villars and K. Cenzual, *Pearson's Crystal Data: Crystal Structure Database for Inorganic Compounds* (ASM International, **2020**).

[6] V. P. Timchenko, A. L. Novozhilov, O. A. Slepysheva, "Kinetics of Thermal Decomposition of Thiourea" *Russ. J. Gen. Chem.* **2004**, *74*, 1046–1050.

[7] T. Li, J. Shi, Z. Liu, W. Xie, K. Cui, B. Hu, G. Che, L. Wang, T. Zhou, C. Liu, "Constructing porous intramolecular donor–acceptor integrated carbon nitride doped with *m* -aminophenol for boosting photocatalytic degradation and hydrogen evolution activity" *Catal. Sci. Technol.* **2022**, *12*, 4591–4604.

[8] Z. Jin, X. Jiang, Q. Zhang, S. Huang, L. Zhang, L. Huang, T. He, H. Zhang, T. Ohno, S. Ruan, Y.-J. Zeng, "Infrared response in photocatalytic polymeric carbon nitride for water splitting via an upconversion mechanism" *Commun. Mater.* **2020**, *1*, 90.

[9] X. Tong, Y. Wu, K. Jiang, J. Jiang, Y. Xu, L. Feng, X. Wang, J. Du, H. Lin, "Oxygen-doped Carbon Nitrides with Visible Room-temperature Phosphorescence and Invisible Thermal-Stimuli-Responsive Ultraviolet Delayed Fluorescence for Security Applications" *Angew. Chem.* **2025**, *137*, e202415312.

[10] S. Zhang, C. Hu, H. Ji, L. Zhang, F. Li, "Facile synthesis of nitrogen-deficient mesoporous graphitic carbon nitride for highly efficient photocatalytic performance" *Appl. Surf. Sci.* **2019**, *478*, 304–312.

[11] Y. Wang, H. Wang, F. Chen, F. Cao, X. Zhao, S. Meng, Y. Cui, "Facile synthesis of oxygen doped carbon nitride hollow microsphere for photocatalysis" *Appl. Catal. B Environ.* **2017**, *206*, 417–425.

[12] L. Zhang, Z. Jin, H. Lu, T. Lin, S. Ruan, X. S. Zhao, Y.-J. Zeng, "Improving the Visible-Light Photocatalytic Activity of Graphitic Carbon Nitride by Carbon Black Doping" *ACS Omega* **2018**, *3*, 15009–15017.

[13] G. Liu, T. Zhang, T. Wang, H. Yamashita, Y. Zhao, X. Qian, "Peroxydisulfate activation by photo-generated charges on mesoporous carbon nitride for removal of chlorophenols" *Appl. Catal. B Environ.* **2021**, *296*, 120370.

[14] J. Hao, W. Liu, Q. Xue, "Effect of N2/CH4 flow ratio on microstructure and composition of hydrogenated carbon nitride films prepared by a dual DC-RF plasma system" *J. Non-Cryst. Solids* **2007**, *353*, 136–142.

[15] H. Liu, Z. Zhu, M. Demir, Y. He, P. Saha, Q. Cheng, "Controllable Synthesis of Heterogeneous ZnS/SnS_2 Encapsulated in Hollow Nitrogen-Doped Carbon Microcubes as Anode for High-Performance Li-ion Capacitors" *Chem. – Asian J.* **2025**, *20*, e202400926.

[16] U. G. M. Ekanayake, K. E. D. Y. T. Dayananda, N. Rathuwadu, M. M. M. G. P. G. Mantilaka, "Fabrication of multifunctional smart polyester fabric via electrochemical deposition of ZnO nano-/microhierarchical structures" *J. Coat. Technol. Res.* **2022**, *19*, 1243–1253.

[17] L. Hu, X. Hu, Z. Lin, Z. Wen, "3D Graphene Network Encapsulating Mesoporous ZnS Nanospheres as High-Performance Anode Material in Sodium-Ion Batteries" *ChemElectroChem* **2018**, *5*, 1552–1558.

[18] A. T. Biradar Tamboli, S. P. Kirdant, V. H. Jadhav, "Metal-free approach towards efficient synthesis of FDCA using a *p* -toluene sulfonic acid (*p* -TSA)-derived heterogeneous solid acid catalyst and oxone over two steps from HMF, fructose and glucose" *New J. Chem.* **2022**, *46*, 10272–10279.

[19] H. Mao, Q. Wu, S. Ma, S. Wu, D. Liu, X. Liu, R. Zhang, "Enhanced nitrate electrosynthesis by *in situ* growth of CoS_2 nanoparticles on poly(zwitterionic liquids) functionalized polypyrrole nanotubes based on accurate nitrate detection" *J. Mater. Chem. A* **2025**, *13*, 40327–40336.

[20] R. Wei, R. Zhang, L. Song, X. Zhou, S. Lin, Y. Zhao, T. Zhou, "Incineration disposal of organic waste bio-residue via a deep dewatering process using refuse incineration bottom ash: moisture transfer and low calorific value improvement" *Environ. Sci. Pollut. Res.* **2022**, *29*, 78107–78119.

[21] Z. Cai, Y. Ou, B. Zhang, J. Wang, L. Fu, M. Wan, G. Li, W. Wang, L. Wang, J. Jiang, Z. W. Seh, E. Hu, X.-Q. Yang, Y. Cui, Y. Sun, "A Replacement Reaction Enabled Interdigitated Metal/Solid Electrolyte Architecture for Battery Cycling at 20 mA cm^{-2} and 20 mAh cm^{-2}" *J. Am. Chem. Soc.* **2021**, *143*, 3143–3152.

[22] Z. Zhao, J. Zhao, Z. Hu, J. Li, J. Li, Y. Zhang, C. Wang, G. Cui, "Long-life and deeply rechargeable aqueous Zn anodes enabled by a multifunctional brightener-inspired interphase" *Energy Environ. Sci.* **2019**, *12*, 1938–1949.

[23] C. Ma, K. Yang, S. Zhao, Y. Xie, C. Liu, N. Chen, C. Wang, D. Wang, D. Zhang, Z. X. Shen, F. Du, "Recyclable and Ultrafast Fabrication of Zinc Oxide Interface Layer Enabling Highly Reversible Dendrite-Free Zn Anode" *ACS Energy Lett.* **2023**, *8*, 1201–1208.

[24] T. Wei, H. Zhang, Y. Ren, L. Mo, Y. He, P. Tan, Y. Huang, Z. Li, D. Zhu, L. Hu, "Building Near-Unity Stacked (002) Texture for High-Stable Zinc Anode" *Adv. Funct. Mater.* **2024**, *34*, 2312506.

[25] Z. Chen, J. Zhao, Q. He, M. Li, S. Feng, Y. Wang, D. Yuan, J. Chen, H. N. Alshareef, Y. Ma, "Texture Control of Commercial Zn Foils Prolongs Their Reversibility as Aqueous Battery Anodes" *ACS Energy Lett.* **2022**, *7*, 3564–3571.

[26] J. Ruan, D. Ma, K. Ouyang, S. Shen, M. Yang, Y. Wang, J. Zhao, H. Mi, P. Zhang, "3D Artificial Array Interface Engineering Enabling Dendrite-Free Stable Zn Metal Anode" *Nano-Micro Lett.* **2023**, *15*, 37.

[27] C. Yang, X. Zhang, J. Cao, D. Zhang, P. Kidkhunthod, S. Wannapaiboon, J. Qin, "Interfacial Reconstruction for Regulating Zn^{2+} Deposition toward Ultrastable Zn Metal Anodes" *ACS Appl. Mater. Interfaces* **2023**, *15*, 26718–26727.

[28] X. Lei, Z. Ma, L. Bai, L. Wang, Y. Ding, S. Song, A. Song, H. Dong, H. Tian, H. Tian, X. Meng, H. Liu, B. Sun, G. Shao, G. Wang, "Porous ZnP matrix for long-lifespan and dendrite-free Zn metal anodes" *Battery Energy* **2023**, *2*, 20230024.

[29] W. Sun, M. Ma, M. Zhu, K. Xu, T. Xu, Y. Zhu, Y. Qian, "Chemical Buffer Layer Enabled Highly Reversible Zn Anode for Deeply Discharging and Long-Life Zn–Air Battery" *Small* **2022**, *18*, 2106604.

[30] D. Yang, J. Li, C. Liu, J. Ge, W. Xing, J. Zhu, "Regulating the MXene–Zinc Interfacial Structure toward a Highly Revisable Metal Anode of Zinc–Air Batteries" *ACS Appl. Mater. Interfaces* **2023**, *15*, 10651–10659.

[31] K. Zhao, C. Wang, Y. Yu, M. Yan, Q. Wei, P. He, Y. Dong, Z. Zhang, X. Wang, L. Mai, "Ultrathin Surface Coating Enables Stabilized Zinc Metal Anode" *Adv. Mater. Interfaces* **2018**, *5*, 1800848.

[32] Y. Wu, Q. Fan, L. Liu, X. Chen, S. Huang, J. Xu, "A Protective Layer of UIO-66/Reduced Graphene Oxide to Stabilize Zinc-Metal Anodes toward High-Performance Aqueous Zinc-Ion Batteries" *ACS Appl. Mater. Interfaces* **2024**, *16*, 34020–34029.

[33] D. Lei, W. Shang, L. Cheng, Poonam, W. Kaiser, P. Banerjee, S. Tu, O. Henrotte, J. Zhang, A. Gagliardi, J. Jinschek, E. Cortés, P. Müller-Buschbaum, A. S. Bandarenka, M. Z. Hussain, R. A. Fischer, "Ion-Transport Kinetics and Interface Stability Augmentation of Zinc Anodes Based on Fluorinated Covalent Organic Framework Thin Films" *Adv. Energy Mater.* **2024**, *14*, 2403030.
[34] C. Wu, K. Xie, K. Ren, S. Yang, Q. Wang, "Dendrite-free Zn anodes enabled by functional nitrogen-doped carbon protective layers for aqueous zinc-ion batteries" *Dalton Trans.* **2020**, *49*, 17629–17634.
[35] L. Xiong, H. Fu, W. Han, M. Wang, J. Li, W. Yang, G. Liu, "Robust ZnS interphase for stable Zn metal anode of high-performance aqueous secondary batteries" *Int. J. Miner. Metall. Mater.* **2022**, *29*, 1053–1060.
[36] A. Kim, H. Kim, K. W. Nam, "Achieving superior stability and cycle life in zinc anodes with aramid surface modification" *J. Mater. Chem. A* **2025**, *13*, 29109–29119.
[37] Q. Cao, Y. Gao, J. Pu, X. Zhao, Y. Wang, J. Chen, C. Guan, "Gradient design of imprinted anode for stable Zn-ion batteries" *Nat. Commun.* **2023**, *14*, 641.
[38] Z. Pan, Q. Cao, W. Gong, J. Yang, Y. Gao, Y. Gao, J. Pu, J. Sun, X. J. Loh, Z. Liu, C. Guan, J. Wang, "Zincophilic 3D ZnOHF nanowire arrays with ordered and continuous Zn2+ Ion modulation layer enable long-term stable Zn metal anodes" *Energy Storage Mater.* **2022**, *50*, 435–443.
[39] L. Kang, M. Cui, F. Jiang, Y. Gao, H. Luo, J. Liu, W. Liang, C. Zhi, "Nanoporous $CaCO_3$ Coatings Enabled Uniform Zn Stripping/Plating for Long-Life Zinc Rechargeable Aqueous Batteries" *Adv. Energy Mater.* **2018**, *8*, 1801090.
[40] J. Zhou, M. Xie, F. Wu, Y. Mei, Y. Hao, R. Huang, G. Wei, A. Liu, L. Li, R. Chen, "Ultrathin Surface Coating of Nitrogen-Doped Graphene Enables Stable Zinc Anodes for Aqueous Zinc-Ion Batteries" *Adv. Mater.* **2021**, *33*, 2101649.
[41] H. He, H. Tong, X. Song, X. Song, J. Liu, "Highly stable Zn metal anodes enabled by atomic layer deposited Al_2 O_3 coating for aqueous zinc-ion batteries" *J. Mater. Chem. A* **2020**, *8*, 7836–7846.
[42] T. Wang, P. Wang, L. Pan, Z. He, L. Dai, L. Wang, S. Liu, S. C. Jun, B. Lu, S. Liang, J. Zhou, "Stabling Zinc Metal Anode with Polydopamine Regulation through Dual Effects of Fast Desolvation and Ion Confinement" *Adv. Energy Mater.* **2023**, *13*, 2203523.
[43] S. Zhou, Y. Wang, H. Lu, Y. Zhang, C. Fu, I. Usman, Z. Liu, M. Feng, G. Fang, X. Cao, S. Liang, A. Pan, "Anti-Corrosive and Zn-Ion-Regulating Composite Interlayer Enabling Long-Life Zn Metal Anodes" *Adv. Funct. Mater.* **2021**, *31*, 2104361.
[44] Y. Wang, X. Lin, L. Wang, Y. Yang, Y. Zhang, A. Pan, "Tailoring the Crystal-Chemical States of Water Molecules in Sepiolite for Superior Coating Layers of Zn Metal Anodes" *Adv. Funct. Mater.* **2023**, *33*, 2211088.
[45] B. Li, B. Zhang, X. Bai, J. Zhang, X. Chang, L. Hou, H. Huang, T. Lu, S. Wang, Z. Jin, Q. Wang, "A Dynamic Self-Healing Protective Layer Enabling Stable Zinc Ion Batteries through Strong Zn-S Affinity and Intramolecular Hydrogen Bonding" *Angew. Chem. Int. Ed.* **2025**, *64*, e202503345.
[46] G. Yoo, Y.-R. Jo, G.-H. An, "Multifunctional Zinc Vanadium Oxide Layer on Metal Anodes Via Ultrathin Surface Coating for Enhanced Stability in Aqueous Zinc-Ion Batteries" *ACS Energy Lett.* **2024**, *9*, 5955–5965.
[47] Y. Yang, C. Liu, Z. Lv, H. Yang, Y. Zhang, M. Ye, L. Chen, J. Zhao, C. C. Li, "Synergistic Manipulation of Zn^{2+} Ion Flux and Desolvation Effect Enabled by Anodic Growth of a 3D ZnF_2 Matrix for Long-Lifespan and Dendrite-Free Zn Metal Anodes" *Adv. Mater.* **2021**, *33*, 2007388.
[48] Z. Mei, H. Li, G. Wang, Y. Mao, Y. Xu, J. Guo, Q. Li, H. Li, W. Li, Y. Tang, X. Liang, "Solvent-free and in situ synthesis of three-dimensional covalent organic frameworks thin films on Zn anodes for Zn–air batteries" *Appl. Surf. Sci.* **2023**, *615*, 156324.
[49] M. Kurian, A. Pandikassala, S. Kurungot, "UV-modified polydopamine zincophilic protective layer for durable and alkaline-stable zinc anodes in zinc–air batteries" *J. Mater. Chem. A* **2025**, *13*, 38928–38944.

[50] Y. Gang, X. Li, L. Liu, X. Cui, J. Yu, Y. Dan, "Highly (002)-oriented ZnO in ZnO-N-C microflakes coating layer for stable zinc anode in zinc-air batteries" *Electrochimica Acta* **2024**, *477*, 143816.
[51] M. A. Deyab, G. Mele, "Polyaniline/Zn-phthalocyanines nanocomposite for protecting zinc electrode in Zn-air battery" *J. Power Sources* **2019**, *443*, 227264.
